# Macrophage polarization in ischemia–reperfusion injury: from molecular mechanisms to therapeutic strategies

**DOI:** 10.3389/fimmu.2026.1826087

**Published:** 2026-04-23

**Authors:** Xiaoyang Han, Aili Zheng, Ping Du, Yixuan Wang, Zhihao Li, Yanbo Hu, Fang Gao, Min Li, Jinmin Guo, Xiaowen Ma

**Affiliations:** 1 School of Pharmacy, Shandong Second Medical University, Weifang, China; 2 The 960th Hospital of People’s Liberation Army (PLA), Jinan, China; 3 Jinzhou Medical University, Jinzhou, China; 4 Shandong Traditional Chinese Medicine University, Jinan, China

**Keywords:** epigenetic regulation, ischemia–reperfusion injury, M1/M2 balance, macrophage polarization, matrix stiffness, metabolic reprogramming

## Abstract

Ischemia–reperfusion injury (IRI) induces secondary tissue damage following the restoration of blood flow. Currently, there is a lack of specific therapeutic interventions for IRI. Macrophage M1/M2 polarization plays a pivotal role in the progression of IRI; however, integrated analyses of its dynamic changes and organ-specific characteristics remain insufficient. This review focuses on the critical role of macrophage M1/M2 phenotypic balance in IRI, systematically elucidating the mechanisms underlying its dynamic regulation. It highlights, for the first time, the impact of metabolic reprogramming and mechanical signaling on polarization imbalance and provides a comprehensive analysis of the organ-specific features of macrophages in four common target organs of reperfusion injury (heart, brain, liver, and kidney). In terms of interventional strategies, cutting-edge approaches are emphasized, including epigenetic drugs, nanoparticle-based targeted delivery systems, and temporally sequenced combination therapies, to achieve precise temporal regulation from suppression of M1-mediated inflammation to promotion of M2-driven repair. In the future, integration of multi-omics and spatiotemporal dynamic analyses will be essential to construct organ-specific and stage-adaptive immune intervention frameworks, thereby advancing the treatment of IRI toward personalization and precision medicine.

## 1 Introduction

The occurrence of ischemia–reperfusion injury (IRI) exhibits paradoxical characteristics, with the restoration of blood flow triggering secondary tissue damage through oxidative stress, calcium overload, and dysregulated immune modulation (1). IRI affects millions of patients worldwide and remains a major contributor to morbidity and mortality (2, 3). Despite substantial advances in reperfusion therapies, such as thrombectomy and angioplasty, IRI-mediated tissue damage remains a critical determinant of patient prognosis (4). Current therapeutic approaches lack specificity, underscoring the urgent need for the development of targeted interventions (5). Among various immune mediators, macrophages orchestrate the biphasic response of IRI through dynamic polarization processes (6). Although the M1/M2 classification paradigm is somewhat simplified, it remains a robust and effective framework for investigating IRI (7). However, when this balance is disrupted, polarization imbalance emerges as the core determinant of tissue injury outcomes (8). Polarization imbalance manifests as persistent M1 dominance or excessive M2 responses, directly determining whether tissues undergo functional recovery or pathological remodeling (9). More importantly, recent single-cell-resolution studies have further revealed organ-specific heterogeneity in macrophages, providing new insights into the complexity of polarization imbalance while challenging the traditional binary M1/M2 classification framework (10). In addition to polarization plasticity, macrophages in IRI are also highly heterogeneous in origin, primarily consisting of tissue-resident macrophages and monocyte-derived macrophages recruited from the circulation. Emerging evidence indicates that these two populations exhibit distinct temporal and functional characteristics across different stages of IRI. Tissue-resident macrophages, which are already present in ischemic tissues prior to reperfusion, are among the first responders to hypoxic stress, whereas monocyte-derived macrophages predominantly contribute to the amplification and resolution of inflammation during later phases. Therefore, understanding the coordinated yet distinct roles of these macrophage subsets is essential for elucidating the complexity of IRI and for developing stage-specific therapeutic strategies (11).

Despite the accumulation of substantial preclinical data on the regulation of IRI by macrophages, three major barriers persist in translating these findings into clinical applications (12). Firstly, understanding of the spatiotemporal dynamics of macrophage polarization remains incomplete, hindering the precise characterization of polarization state transitions across different phases and tissue regions (13). Secondly, the absence of organ-specific intervention strategies limits the development of tailored approaches that account for macrophage heterogeneity in distinct affected organs, particularly during the ischemic and early reperfusion phases, when inflammatory responses and tissue damage are most pronounced (14, 15). Thirdly, the lack of validated biomarkers for patient stratification prevents the effective identification of individuals most likely to benefit from macrophage-targeted therapies. Specifically, patient stratification could be based on parameters such as inflammatory cytokine levels, and the degree of tissue damage during ischemia-reperfusion injury (16–18). Addressing these challenges requires resolution of key controversies in macrophage functional regulation, such as the dual role of M2 macrophages, which exert tissue-protective effects during the acute phase of IRI but may promote fibrosis in the chronic phase. At present, the mechanisms underlying the stage-dependent and bidirectional nature of this functionality have not been elucidated. Thus, breakthroughs in this area would provide critical theoretical support for optimizing subsequent intervention strategies (19).

Considering the translational challenges in macrophage-mediated IRI regulation, this review systematically integrates the molecular mechanisms governing macrophage polarization in cardiac, cerebral, hepatic, and renal IRI (20). The regulatory mechanisms are examined across five key regulation dimensions, namely transcriptional (21), metabolic (22), epigenetic (23), mechanical signaling (24), and metabolite signaling (25). Emerging therapeutic modalities are critically evaluated (26, 27) to delineate their potential and limitations in different organ-specific IRI contexts. The ultimate goal of this approach is to formulate immune modulation strategies that are both organ-specific and phase-adaptive by integrating the distinct pathological stages of IRI with the unique characteristics of affected organs, thereby providing directional guidance to overcome the key obstacles previously encountered in clinical translation.

## 2 Macrophage polarization phenotypes and functional transitions

Macrophages undergo dynamic phenotypic and functional changes in response to microenvironmental cues (28). They are conventionally classified into pro-inflammatory M1 and anti-inflammatory/reparative M2 subtypes based on cytokine secretion and surface markers (29). The balance between M1 and M2 phenotypes critically determines the direction of the immune response and tissue recovery in conditions such as IRI (6). Notably, macrophages exhibit pronounced spatiotemporal heterogeneity across different organs and temporal phases (13). Their phenotypes and functions are finely orchestrated by organ-specific signals, metabolic states, and microenvironmental fluctuations (30). Thus, understanding the molecular hubs, metabolic profiles, and signaling mechanisms of M1/M2 polarization is crucial for elucidating IRI and guiding precision interventions (13, 31).

### 2.1 M1/M2 phenotype balance: the cornerstone and regulatory key of IRI immunopathology

M1 macrophages drive the production of cytokines such as TNF-α, IL-1β, and IL-6, as well as the release of ROS and nitric oxide (NO), through the nuclear factor-κB/mitogen-activated protein kinase (NF-κB/MAPK) signaling pathways, thereby amplifying the inflammatory response (32, 33). Although this process is essential for pathogen clearance and the removal of necrotic cells, excessive M1 activation sustains endothelial injury and apoptosis, establishing a pro-inflammatory amplification loop (34). In contrast, M2 macrophages are induced during the resolution phase by stimuli such as IL-4, IL-13, and IL-10, leading to the secretion of anti-inflammatory mediators, including IL-10 and TGF-β. These effects suppress excessive inflammation and promote extracellular matrix remodeling and cellular regeneration (35–37). However, prolonged M2 responses may culminate in fibrosis and loss of tissue function (38–41). Based on distinct activating signals and functional profiles, M2 macrophages are further subdivided into M2a, M2b, M2c, and M2d subsets, which exhibit partial overlap in surface markers and functions (42). Among these, the characteristics and regulatory mechanisms of the M2b subset remain to be fully elucidated (43, 44).

### 2.2 Phase-specific M1/M2 transitions: spatiotemporal regulation of the inflammation–repair balance

The advent of single-cell RNA sequencing has challenged the traditional M1/M2 dichotomy, further revealing that M1 and M2 phenotypes undergo precise temporal transitions and functional coupling during injury and repair processes (45–47). These “phase-specific” polarization dynamics govern the initiation and resolution of inflammation as well as the onset and outcome of tissue repair. Therefore, they constitute the cornerstone for understanding the immunopathophysiological core of IRI and implementing temporally targeted interventions (14, 48).

During the early phase of IRI (0–24 h), acute hypoxia and initial tissue damage dominate the local microenvironment (49). Damage-associated molecular patterns (DAMPs) released from necrotic cells, such as high mobility group box 1 protein (HMGB1) and adenosine triphosphate (ATP), activate macrophage surface toll-like receptors 2/4 (TLR2/4) and P2X purinergic receptor 7 (P2X7) (50), thereby rapidly triggering the NF-κB and MAPK signaling pathways as well as the NOD-like receptor family pyrin domain-containing 3 (NLRP3) inflammasome (51). Concurrently, hypoxia-inducible factor-1α (HIF-1α) drives metabolic reprogramming in macrophages toward a highly glycolytic phenotype (52). In synergy with the Notch1 and signal transducer and activator of transcription 1 (STAT1) signaling pathways, this process promotes preferential polarization of macrophages toward the M1 phenotype (53, 54). At this stage, M1 macrophages prioritize phagocytic clearance of necrotic tissue debris and potential pathogens, while releasing pro-inflammatory mediators (e.g., ROS/NO, TNF-α, IL-1β, and IL-6) to accomplish pathogen elimination and antigen presentation. These effects intercept DAMP leakage and inflammatory amplification at the source (55–57).

Concomitantly, a minor population of tissue-resident or early-recruited M2 cells secretes IL-10 and TGF-β, exerting early suppression of NF-κB via the Janus kinase/STAT3 (JAK/STAT3) pathway, thereby preserving pathways for subsequent tissue repair programs (58–60). In case the intensity and duration of the pro-inflammatory response in this phase become dysregulated, the phagocytic clearance function of M1 macrophages may become inverted relative to their pro-inflammatory secretory function, allowing residual DAMPs to persistently stimulate the TLR/NF-κB axis. This results in consolidation of the pro-inflammatory loop and ultimately impedes the initiation of repair programs (61, 62).

In the intermediate phase of IRI (approximately 24–72 h), as reperfusion restores blood flow and local oxygen tension rises, inflammation enters a critical “contention period” characterized by the coexistence of peak inflammatory activity and transitional turning points. On one hand, M1 macrophages maintain dominance under the drive of chemokines such as IL-1β, C-X-C motif chemokine ligand 8 (CXCL8), and C-C motif chemokine ligand 2 (CCL2) (63), continuously recruiting neutrophils and C-C chemokine receptor type 2 (CCR2)-positive (CCR^2+^) monocytes (64). The STAT1/mammalian target of rapamycin complex 1 (STAT1/mTORC1) signaling axis further reinforces glycolytic metabolism and pro-inflammatory transcription, establishing an amplification loop of recruitment, polarization, and renewed recruitment (65–67). Concurrently, ATP-P2X7-mediated calcium influx and potassium efflux sustain NLRP3 inflammasome activation, thereby prolonging the injury window (68, 69). On the other hand, reoxygenation attenuates HIF-1α-dependent signaling, while the IL-4/IL-13 and IL-10/STAT3 pathways are progressively upregulated, resulting in an increase in M2 macrophage numbers (70–73). During this period, M2 macrophages suppress pro-inflammatory cascades and sustain neutrophil infiltration via IL-10/TGF-β, secrete matrix metalloproteinase-2 (MMP-2) and MMP-9, and release angiogenic and mitogenic factors such as vascular endothelial growth factor (VEGF) and platelet-derived growth factor (PDGF) to promote neovascularization and parenchymal cell proliferation. These effects facilitate the transition from inflammation to resolution and repair (74, 75). If synchronized downregulation of M1 and upregulation of M2 phenotypes can be achieved within this temporal window, inflammation successfully shifts toward resolution and tissue reconstruction. Conversely, persistent M1 dominance coupled with high DAMP burden establishes a mutually reinforcing vicious cycle, leading to expansion of the lesion and diffusion of collateral tissue damage (76–78).

In the late phase of IRI (>72 h), under optimal conditions, the number and activity of M1 macrophages gradually decline, allowing an M2-dominated immune-regenerative network to maintain a low-inflammatory milieu that promotes granulation tissue maturation, orderly vascular and matrix reconstruction, and ultimately organ functional recovery (79). This process is sustained by critical drivers, including the Wnt/β-catenin pathway, STAT6, and oxidative phosphorylation/fatty acid oxidation (OXPHOS/FAO) metabolism (80). However, imbalance in either direction delays repair. Specifically, persistent residual M1 activity and low-level TNF-α/IL-6 can sustain chronic inflammatory tension via the TLR/NF-κB and iNOS/NO axes, suppressing IL-10/TGF-β-driven remodeling programs and resulting in a chronic state of “refractory inflammation and delayed repair.” Conversely, excessive or prolonged M2 activation leads to sustained TGF-β secretion and matrix synthesis, escalating collagen deposition and sclerosis, thereby markedly increasing the risk of myocardial, hepatic, and renal interstitial fibrosis (61). Therefore, temporally phased immunotherapy is particularly critical. In the advanced stage, it is advisable to suppress residual pro-inflammatory inputs while restraining excessive reparative processes, and, in coordination with organ-specific pathways, improve immune resolution and functional reconstruction (81–84).

**Figure 1:**
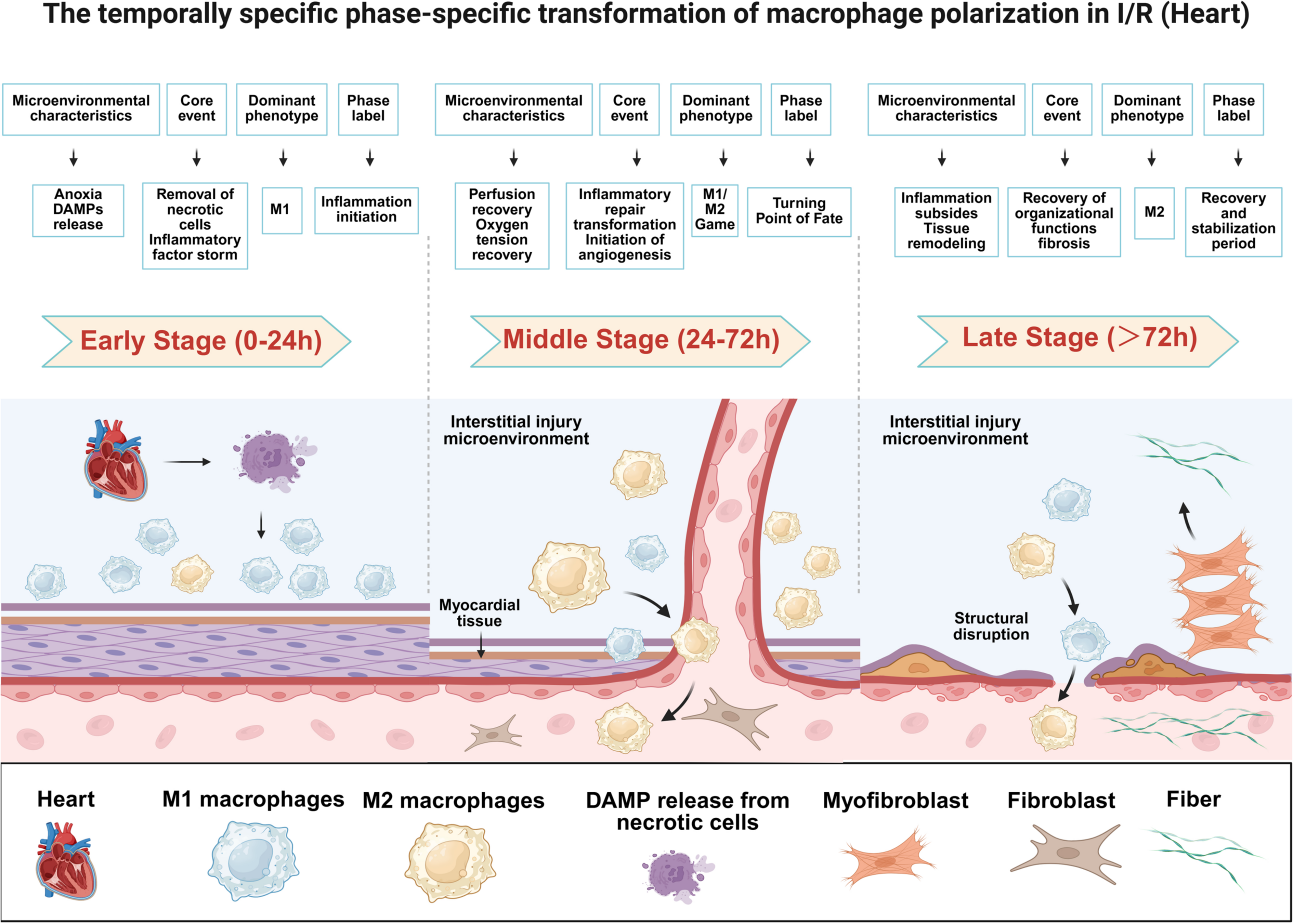
Temporal-specific transitions in macrophage polarization during cardiac ischemia–reperfusion injury. The heart icon denotes the target organ undergoing ischemia–reperfusion injury. The purple cluster in the early stage represents necrotic cardiomyocyte debris and the release of damage-associated molecular patterns (DAMPs). Light blue cells indicate pro-inflammatory M1 macrophages, whereas pale yellow cells indicate anti-inflammatory/reparative M2 macrophages. The orange spindle-shaped cells represent myofibroblasts, the brown stellate cells represent fibroblasts, and the green wavy structures indicate collagen fibers. The purple layered band represents injured myocardial tissue, and the space above it denotes the interstitial injury microenvironment. Arrows indicate key sequential events, including ischemic injury, DAMP release, macrophage recruitment and polarization, M1-to-M2 transition, tissue remodeling, and fibrosis. Progressive thinning and focal disruption of the myocardial layer reflect ongoing cardiomyocyte loss, inflammatory injury, and extracellular matrix degradation during early-to-middle stages, whereas the late stage is characterized by M2-dominant repair, structural remodeling, and possible fibrotic deposition. DAMPs, damage-associated molecular patterns; I/R, ischemia–reperfusion.

Overall, M1- and M2-like macrophages exhibit complementary and dynamically balanced polarization processes throughout the early, intermediate, and late stages of IRI. Importantly, the regulatory roles of these signaling pathways are highly stage-dependent. In the early phase (0–24 h), pro-inflammatory pathways, such as TLR2/4–NF-κB signaling, contribute to the rapid initiation of innate immune responses and may facilitate the clearance of damaged cells (20, 85). However, excessive or sustained activation of these pathways can exacerbate tissue injury (85). During the intermediate phase, a shift toward anti-inflammatory and reparative signaling becomes critical. Pathways promoting M2-like polarization, including STAT6 and PI3K/Akt signaling, help resolve inflammation and initiate tissue repair (86, 87). Notably, the duration and relative importance of these pathways vary across organs due to distinct microenvironmental contexts. Therefore, precise temporal modulation of these pathways is essential for optimizing therapeutic outcomes in IRI. Disruption of this balance may lead to either persistent inflammation or excessive repair. Future studies should focus on defining and targeting the immune transition phase, which may offer greater clinical value than simply enhancing a single macrophage phenotype. (**Figure 1**).

### 2.3 Differential roles of tissue-resident and monocyte-derived macrophages in IRI

Furthermore, macrophage origin represents an important additional layer of regulation in IRI. Macrophages involved in IRI are derived from two major sources: tissue-resident macrophages and infiltrating monocyte-derived macrophages (88, 89). These two populations exhibit distinct temporal dynamics and functional properties, which play complementary roles in initiating and resolving inflammation during different stages of IRI.

Tissue-resident macrophages are present in organs prior to ischemia and are among the earliest immune cells to respond to hypoxic stress (90). During the ischemic phase and early reperfusion, these cells rapidly respond to microenvironmental changes by sensing danger-associated molecular patterns (DAMPs) and releasing inflammatory mediators. In this early stage, resident macrophages play a key role in initiating the inflammatory cascade and shaping the local immune response. In contrast, monocyte-derived macrophages are recruited from the circulation following reperfusion, and their accumulation becomes more prominent during the later phases of IRI (91). These macrophages contribute to the amplification of inflammation, as well as tissue repair and remodeling. Depending on the specific microenvironment, these recruited macrophages may exhibit either pro-inflammatory or pro-resolving phenotypes, thereby playing a dual role in the progression and recovery of the injury (20).

Importantly, the relative contribution of these two macrophage populations varies across organs and stages of IRI. For example, in the liver, Kupffer cells (resident macrophages) are key initiators of early inflammatory responses (6), whereas in the kidney and heart, both resident and monocyte-derived macrophages contribute dynamically at different stages of IRI (7, 92). Overall, resident macrophages are more critical in the early phase of ischemia and reperfusion, while monocyte-derived macrophages become increasingly important during the progression and resolution phases. These findings highlight the necessity of considering macrophage heterogeneity in both temporal and spatial contexts when interpreting their roles in IRI.

## 3 Biochemical signaling mechanisms regulating macrophage polarization in IRI

### 3.1 Intracellular signaling pathway networks

#### 3.1.1 NF-κB pathway

NF-κB serves as the core transcription factor driving M1 polarization, and its activation depends on the canonical signaling cascades mediated by TLR4/myeloid differentiation primary response protein 88 (TLR4/MyD88), tumor necrosis factor receptor 1 (TNFR1), and interleukin-1 receptor (IL-1R) (93, 94). In the immune response to IRI, DAMPs released from necrotic cells and pro-inflammatory cytokines activate the inhibitor of κB kinase (IKK) complex via the aforementioned receptors, thereby initiating the NF-κB pathway. This leads to nuclear translocation of the p65/p50 heterodimer and subsequent transcription of pro-inflammatory genes encoding TNF-α, IL-1β, IL-6, and others, thus sustaining the pro-inflammatory activation state of macrophages (95, 96). NF-κB activation exhibits a stage-dependent dual role in IRI. In the early phase, NF-κB activation contributes to the rapid initiation of innate immune responses and facilitates the clearance of damaged cells, thereby exerting protective effects (97, 98). However, sustained or excessive NF-κB activation in later stages prolongs and amplifies inflammation while suppressing pro-reparative signaling mediated by PPAR-γ and STAT6, ultimately impairing M2 polarization and tissue repair (99–101). Thus, NF-κB functions not only as a central regulator of M1 polarization but also as a key determinant of stage-dependent inflammatory outcomes in IRI. Studies have demonstrated that activation of nuclear factor erythroid 2-related factor 2 (Nrf2) antagonizes NF-κB-mediated pro-inflammatory effects, attenuating M1 polarization and reducing inflammatory cytokine release. In murine IRI models, Nrf2 knockout or administration of its specific inhibitor (ML385) exacerbates renal injury (102, 103). In addition, clinical observations revealed a positive correlation between the degree of NF-κB overactivation and the severity of IRI tissue damage (31). These findings establish NF-κB as one of the primary drivers of polarization imbalance and sustained inflammation during IRI progression.

#### 3.1.2 JAK/STAT signaling axis

The JAK/STAT pathway serves as the central hub for cytokine-mediated immune responses, exhibiting bidirectional regulatory characteristics (104, 105). In IRI, dysregulation of this pathway manifests as concurrent amplification of pro-inflammatory signals and suppression of anti-inflammatory signals. Interferon-γ (IFN-γ) activates the JAK1/2/STAT1 pathway, driving gene transcription in M1 macrophages and promoting the release of pro-inflammatory factors such as TNF-α and IL-1β, thereby intensifying immune responses and exacerbating tissue damage (106, 107). In contrast, IL-4 and IL-13 activate the JAK1/3/STAT6 pathway to induce M2 polarization, thus initiating anti-inflammatory and tissue repair programs (108).

However, in the pathological milieu of IRI, this dynamic equilibrium is disrupted by multiple factors. Excessive STAT1 activation further exacerbates M1 polarization and pro-inflammatory responses, while oxidative stress and epigenetic modifications significantly inhibit STAT6 activity. These effects impede IL-4/IL-13-induced M2 polarization and hinder the establishment of tissue repair and immune tolerance (109, 110). This dual imbalance, characterized by enhanced pro-inflammatory signaling and blocked repair signals, prolongs the duration of inflammation and exacerbates the degree of tissue damage. At the upstream regulatory level, a study detected high expression of triggering receptor expressed on myeloid cells 2 (TREM2) in renal IRI tissues, predominantly localized to macrophages (111). TREM2 deficiency suppresses the mammalian target of rapamycin (mTOR) pathway and downregulates suppressors of cytokine signaling 1/3 (SOCS1/3), leading to hyperactivation of JAK/STAT, enhanced M1 polarization, and aggravated renal injury. These observations suggest that targeting TREM2 upregulation improves the pro-reparative functions of macrophages (112). Another investigation demonstrated that IL-10 negatively regulates inflammatory responses via STAT3, typically alleviating immune dysregulation in IRI (113, 114); however, in environments characterized by persistent DAMP accumulation and excessive ROS production, STAT3 activity is often suppressed, impeding timely reversal of the inflammatory state and occasionally exacerbating inflammatory propagation due to imbalance (115, 116). Thus, dysregulation of the JAK/STAT signaling axis constitutes a key mechanism underlying immune imbalance in IRI, with its bidirectional regulatory properties offering potential therapeutic targets for restoring immune equilibrium.

#### 3.1.3 Phosphatidylinositol 3-kinase/protein kinase B/mTOR signaling axis

The PI3K/AKT/mTOR axis constitutes a pivotal component of the regulatory network governing macrophage polarization (117, 118). Enhanced activity of downstream mTORC1 is frequently associated with reinforcement of the pro-inflammatory M1 phenotype, promoting the production of inflammatory mediators and augmenting phagocytic and bactericidal functions (119, 120). Moreover, intact mTORC2 signaling supports the anti-inflammatory and reparative functions of M2 macrophages, thereby facilitating the expression of anti-inflammatory cytokines (e.g., IL-10) and tissue repair-associated genes (121, 122). In the pathological context of IRI, dysregulation of the PI3K/AKT/mTOR pathway becomes particularly pronounced. Hyperactivation of PI3K/AKT/mTOR signaling sustains macrophages in a prolonged M1 pro-inflammatory state, leading to persistent high-level expression of pro-inflammatory mediators and continuous recruitment of immune cells, thereby exacerbating inflammatory amplification and tissue damage (123, 124). Concurrently, factors such as oxidative stress impair mTORC2-mediated anti-inflammatory signaling, obstructing M2 polarization and compromising the tissue-reparative capacity of macrophages, thus establishing a state of enhanced pro-inflammatory drive coupled with blocked reparative signaling (124, 125). Notably, the PI3K/AKT/mTOR pathway interacts synergistically with other inflammatory signaling cascades, including NF-κB and STAT3, during the modulation of macrophage function (126). In a retinal IRI model, Rhein-GFFYE (Rh-GFFYE) nanofibers that simultaneously activate the PI3K/AKT/mTOR pathway while inhibiting NF-κB/STAT3 signaling were developed, achieving coordinated regulation of multiple axes. This finding underscores the central role of the PI3K/AKT/mTOR axis in inflammatory responses and polarization balance and provides a novel paradigm for multi-pathway synergistic intervention in IRI (127).

#### 3.1.4 Notch and Wnt/β-catenin signaling axes

The Notch1/recombination signal binding protein for immunoglobulin kappa J region (Notch1/RBP-Jκ) axis represents another critical regulator of M1 polarization (128). During IRI, abundant DAMPs and pro-inflammatory cytokines synergistically enhance Notch signaling activity. This leads to elevated macrophage expression of inducible nitric oxide synthase (iNOS), TNF-α, and other pro-inflammatory molecules, thereby exacerbating M1 polarization and tissue damage. Excessive Notch pathway activation further sustains and intensifies inflammatory responses (129, 130). In contrast, the Wnt/β-catenin pathway functions as a protective mechanism by activating β-catenin to promote M2 macrophage polarization, upregulating the expression of reparative genes (e.g., IL-10 and Arg1) and enhancing anti-inflammatory and tissue regenerative capabilities (131, 132). In IRI, hyperactivation of Notch signaling coupled with suppression of Wnt signaling is commonly observed, resulting in dysregulation of pro-inflammatory and reparative control. Excessive Notch activity prolongs the M1 polarization state and amplifies inflammatory mediator release, while impaired Wnt signaling restricts M2 polarization and repair processes. Collectively, these effects drive inflammatory expansion and impede tissue regeneration (132, 133). Therefore, inhibiting excessive Notch signaling while restoring Wnt pathway function is a promising strategy for the amelioration of immune imbalance in IRI. In-depth investigation of the interactions between the Notch and Wnt pathways in IRI may facilitate the development of more precise immunomodulatory therapies (132–135).

**Figure 2:**
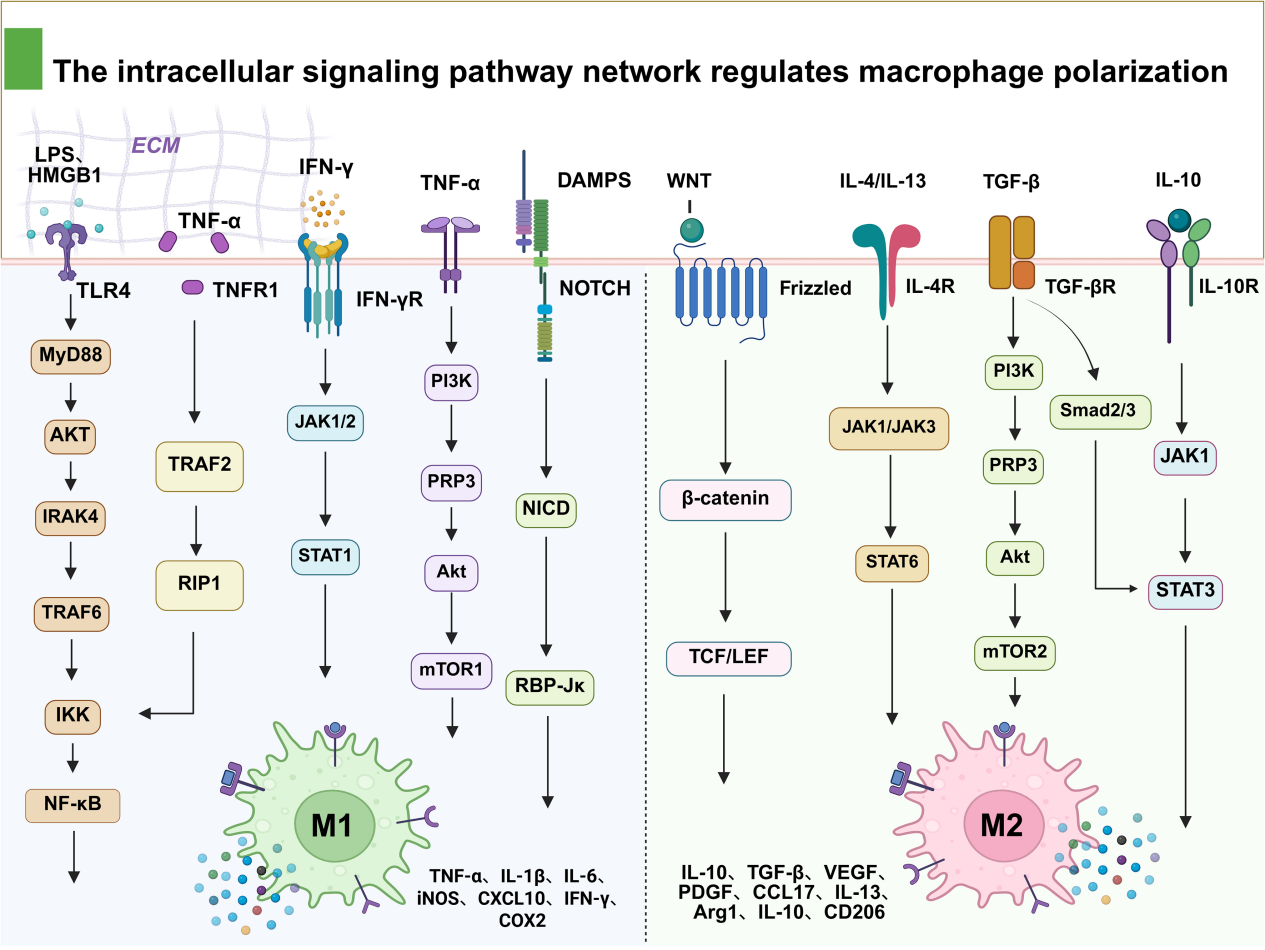
Intracellular signaling pathway map of IRI-associated macrophage M1/M2 phenotypic switching. The green and pink cells are M1 and M2 macrophages, respectively. On the left, LPS/HMGB1, IFN-γ, etc. are regulated by TLR4, IFN-γR, etc. receptors through the MyD88 and JAK1/2-STAT1 pathways, ultimately producing pro-inflammatory molecules such as TNF-α and iNOS. On the right, IL-4/IL-13, TGF-β, etc. are regulated by IL-4R, TGF-βR, etc. receptors through β-catenin and JAK-STAT6 pathways, generating repair-related molecules such as Arg1 and CD206. DAMPs, damage-associated molecular patterns; ECM, extracellular matrix; IRI, ischemia–reperfusion injury; LPS, lipopolysaccharide.

To address the macrophage polarization imbalance in IRI, future interventional strategies should leverage the dynamic coupling characteristics of pro-inflammatory and reparative signaling axes, employing phased and finely tuned regulatory approaches. In the early stage of IRI, priority should be given to suppressing pro-inflammatory pathways (e.g., NF-κB, STAT1, mTORC1, and Notch) to prevent excessive immune responses. Subsequently, within appropriate temporal windows, the activation of reparative pathways including STAT6, STAT3, Wnt/β-catenin, and Nrf2 should be promoted to facilitate M2 polarization and tissue repair (136–138). This temporally sequenced, multi-pathway combined intervention paradigm is expected to restore immune system equilibrium, substantially enhance the precision and efficacy of IRI treatment, and ultimately improve long-term patient prognosis (**Figure 2**).

### 3.2 Extracellular signals and receptor activation

#### 3.2.1 Persistent release of DAMPs and receptor activation

During IRI, extensive necrotic cell death releases abundant DAMPs, including HMGB1, ATP, and heat shock proteins (HSPs). These rapidly bind to pattern recognition receptors on the macrophage surface, triggering robust pro-inflammatory signaling cascades (139). Specifically, HMGB1 and HSPs activate TLRs (e.g., TLR2 and TLR4), whereas ATP acts as a danger signal through the P2X7 ion channel receptor, initiating downstream MyD88/interleukin-1 receptor-associated kinase 4/tumor necrosis factor receptor-associated factor 6-dependent (MyD88/IRAK4/TRAF6-dependent) NF-κB/MAPK pathways (140, 141). These cascades drive rapid high-level expression of pro-inflammatory cytokines and activate the NLRP3 inflammasome. This promotes the maturation and release of pro-IL-1β, thereby shifting macrophages toward the M1 phenotype (142–144). Transient stimulation of low levels of DAMPs enhances the capacity of macrophages to clear pathogens and necrotic debris; however, sustained secondary cell death during reperfusion leads to prolonged accumulation of high levels of DAMPs, resulting in persistent hyperactivation of the TLR/NF-κB pathway (145). This sustains the elevated release of pro-inflammatory mediators (e.g., TNF-α and IL-1β) and suppresses anti-inflammatory signals mediated by PPAR-γ and STAT6. These effects disrupt the transition from the M1 to M2 phenotype and block progression from inflammation to repair (146). Additionally, excessive extracellular ATP induces cytosolic Ca^2+^ influx and K^+^ efflux via P2X7 receptors. This serves as a second signal for the assembly of the NLRP3 inflammasome and markedly enhances the caspase-1-mediated maturation and secretion of IL-1β, further amplifying the pro-inflammatory effects (147, 148). Consequently, M1 macrophages predominate and fail to transition to the anti-inflammatory and reparative M2 phenotype.

Notably, peroxiredoxin-1 (Prdx1) has recently been identified as a critical DAMP in IRI, and its receptor-mediated pathway in macrophages reinforces the aforementioned regulatory mechanisms. In models of renal IRI and sepsis-associated acute kidney injury, deficiency leads to necrosis of proximal tubular epithelial cells and massive Prdx1 release. Prdx1 specifically binds the macrophage-inducible C-type lectin receptor (Mincle) on macrophages. This binding activates the downstream spleen-associated tyrosine kinase/NF-κB (Syk/NF-κB) signaling cascade, promoting the polarization of macrophages toward the M1 phenotype and substantially increasing the production of pro-inflammatory cytokines including IL-1β, IL-6, and TNF-α. The outcome of this process is aggravated tubular necrosis, renal dysfunction, and amplified local inflammation. Multiple studies have validated the pro-inflammatory role of this “Prdx1/Mincle” axis from various perspectives (149). Therefore, reducing the circulating Prdx1 levels or blocking its interaction with Mincle holds promise for disrupting DAMP-driven inflammatory amplification loops, restoring M1/M2 polarization balance in injured tissues, and offering novel therapeutic targets for anti-inflammatory interventions in IRI (149, 150).

#### 3.2.2 Synergy between oxidative stress and inflammatory signaling

Reperfusion-induced oxidative stress likewise plays a pivotal role in macrophage polarization imbalance. In the early reperfusion phase, a transient surge in ROS can, to a certain extent, enhance the capacity of M1 macrophages to clear necrotic debris by activating pathways such as Nrf2 and MAPK (88, 151). However, excessive ROS production damages mitochondria and suppresses key transcription factors essential for M2 polarization. These effects prevent the transition of macrophages from a glycolysis-dominated pro-inflammatory metabolic profile to an OXPHOS-predominant anti-inflammatory metabolic state (88). The direct consequence of this process is prolonged M1 dominance, accompanied by delayed initiation of M2-mediated anti-inflammatory and reparative programs. Sustained high levels of ROS extend the inflammatory window and indirectly delay the transition from inflammation to resolution by impairing metabolic reprogramming in macrophages. Of note, there is synergistic interplay between inflammatory signaling and dysregulated oxygen metabolism. Activation of the NF-κB pathway by pro-inflammatory signals within macrophages stabilizes HIF-1α, thereby sustaining the transcription of pro-inflammatory genes even after oxygen supply is restored, effectively maintaining macrophages in the M1 state and prolonging the inflammatory phase (152, 153). Studies have confirmed that pathogen- or DAMP-induced NF-κB activation reinforces HIF signaling by inducing HIF-1α expression, ensuring persistence of the pro-inflammatory program within the inflammatory microenvironment (154). Furthermore, endoplasmic reticulum stress triggers mitochondrial Ca^2+^ overload and excessive ROS generation, which in turn activates the NLRP3 inflammasome, amplifying M1 polarization and tissue injury. Li et al. identified this “endoplasmic reticulum stress–mitochondrial Ca^2+^–ROS/NLRP3” axis as a central mechanism driving pro-inflammatory macrophage activation and tissue damage in a fatty liver IRI model (155, 156).

#### 3.2.3 Spatiotemporal dysregulation of the chemokine network

The spatiotemporal dysregulation of the chemokine-receptor network likewise constitutes a critical driver of macrophage polarization bias. The chemokine gradients within the injured microenvironment profoundly influence the trajectory of post-IRI inflammation and repair by governing the directed recruitment, retention, and differentiation of monocytes/macrophages (157, 158). Following the onset of IRI, a pro-inflammatory chemokine profile rapidly emerges at the site of injury, characterized by marked upregulation of CCL2, CCL3, CXCL1, CXCL8, and related factors. These chemokines engage CCR2, CCR5, and CXCR2 on macrophages and monocytes, continuously mobilizing Ly6Chigh CCR^2+^ inflammatory monocytes across the damaged vascular endothelium into the injured tissue (157). In an inflammatory milieu rich in DAMPs, TNF-α, and IL-1β, newly infiltrated monocytes preferentially differentiate into M1 macrophages, intensifying local pro-inflammatory effects. Moreover, CCL2 secreted by M1 macrophages further activates the CCR2 axis, establishing a self-reinforcing cycle of sustained recruitment, biased differentiation, and renewed recruitment that progressively amplifies inflammatory monocyte–macrophage infiltration and M1 polarization (157). Animal models and clinical studies demonstrated that persistently hyperactive CCL2/CCR2 signaling in renal and myocardial IRI strongly correlates with massive M1 macrophage infiltration and elevated CCL2 gene expression, with axis activity sustained by positive feedback from NF-κB signaling (159, 160).

**Figure 3:**
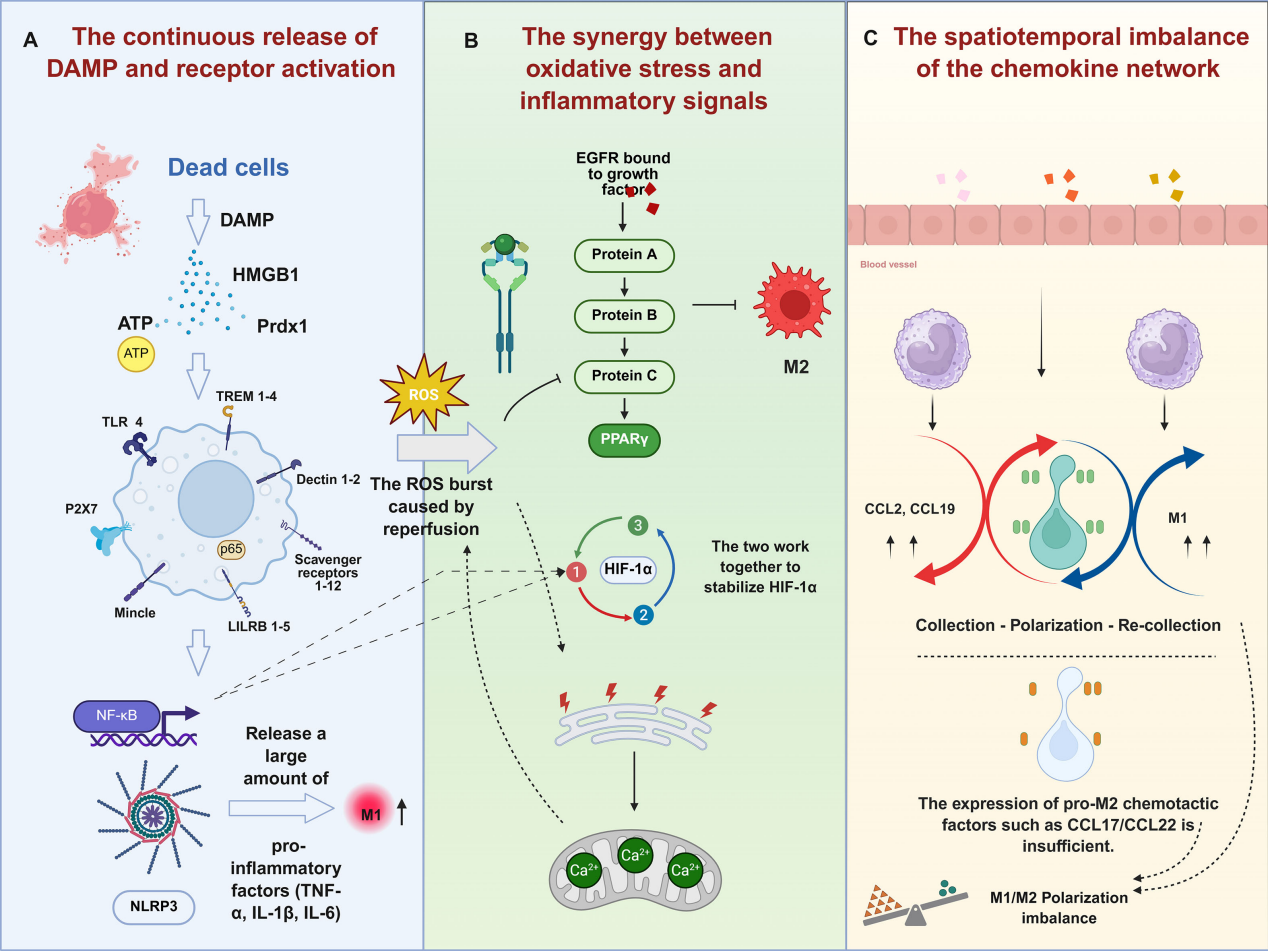
Molecular pathway map of macrophage polarization mediated by three major extracellular mechanisms in IRI. **(A)** Dead cells release DAMPs (e.g., HMGB1, ATP), which bind to TLR4/TREM and other receptors on damaged intestinal epithelial cells, activate the NF-κB/NLRP3 pathway, and promote the generation of M1 macrophages as well as the production of pro-inflammatory factors. **(B)** The reperfusion process triggers an oxidative burst, which stabilizes HIF-1α through the EGFR pathway and PPAR-γ in a coordinated manner, simultaneously affecting calcium signal transduction and participating in the regulation of M2-related processes. **(C)** Chemokines at the vascular site (e.g., CCL2) mediate the “recruitment - conformational conversion - re-recruitment” cycle. However, the expression of pro-M2 macrophage chemokines (CCL17/22) is insufficient, resulting in an imbalance in the M1/M2 conformational conversion. DAMPs, damage-associated molecular patterns; IRI, ischemia–reperfusion injury.

In contrast to the robust activation of pro-inflammatory chemokine signals, reparative M2-associated chemotactic cues are often insufficient and disorganized. M2-favorable chemokines such as CCL17, CCL18, and CCL22 are expressed at low levels, failing to effectively mobilize anti-inflammatory monocytes from distant sites. In addition, endothelial injury disrupts the CXCL12/CXCR4 homeostatic gradient, thereby depriving M2 macrophages of clear navigational signals and preventing their timely accumulation at injury margins (161). Simultaneously, massive infiltration of neutrophils and inflammatory monocytes driven by the CXCR2 axis occupies matrix-binding sites and alters the local microenvironment, further attenuating reparative chemokine gradients (162, 163). Additionally, the CX3CL1/CX3CR1-mediated recruitment of resident/reparative monocytes is compromised by microvascular damage; this results in inability to compensate for the functional deficit in circulating monocyte mobilization (164). This imbalance in the chemotactic network ultimately prolongs M1 dominance, rendering replacement by M2-mediated resolution and tissue remodeling difficult. Consequently, inflammation resolution and injury repair are markedly delayed, accompanied by a substantially elevated risk of fibrosis and organ dysfunction (**Figure 3**).

### 3.3 Metabolic reprogramming

#### 3.3.1 Glycolysis and the tricarboxylic acid cycle

The “incomplete operation” of the mitochondrial TCA cycle is a prominent feature of the metabolic reprogramming of classical M1 macrophages. This cycle is blocked at two key nodes, namely isocitrate dehydrogenase (IDH) and succinate dehydrogenase (SDH). This blockade directly leads to the accumulation of upstream metabolite citrate and downstream metabolite succinate in large quantities (165–167). Excess citrate is transported to the cytosol via mitochondrial carriers and cleaved by ATP-citrate lyase (ACLY) into acetyl-coenzyme A (acetyl-CoA) and reduced nicotinamide adenine dinucleotide phosphate (NADPH). This process provides the essential substrates and reduces inflammatory mediator synthesis. Acetyl-CoA serves as a precursor for lipid inflammatory mediators such as prostaglandins, whereas NADPH supports the activity of iNOS and NADPH oxidase (NOX), thereby promoting NO and ROS generation (168–170). Concurrently, accumulated succinate assumes a signaling molecule-like role in M1 macrophages. Elevated intracellular succinate inhibits prolyl hydroxylase (PHD), stabilizing HIF-1α and driving transcription of pro-inflammatory genes such as IL-1β even under normoxic conditions (171). Excess succinate also triggers reverse electron transport at mitochondrial complex II, thus generating large amounts of ROS and further intensifying inflammation (172). Notably, macrophages can export surplus succinate extracellularly, where it engages the G protein-coupled receptor succinate receptor 1 (SUCNR1; GPR91) in an autocrine/paracrine manner. This leads to activation of the downstream extracellular regulated kinase/MAPK (ERK/MAPK) and NF-κB pathways to amplify pro-inflammatory signaling (173, 174). Thus, succinate functions as a metabolite and a key pro-inflammatory mediator; its overaccumulation drives polarization toward the M1 phenotype and establishes a positive inflammatory feedback loop, perpetuating inflammation and exacerbating tissue injury (175). In diseases such as atherosclerosis and obesity, local succinate elevation promotes macrophage infiltration and sustains the M1 phenotype via SUCNR1 (176–178). Moreover, in hepatic IRI models, SUCNR1-deficient mice exhibited markedly attenuated M1 polarization and reduced inflammatory damage (179). This body of evidence identified the succinate/SUCNR1 axis as a major driver of pro-inflammatory macrophage polarization.

Contrary to the pro-inflammatory effect of succinic acid, itaconate (another product of macrophage metabolic reprogramming) plays an endogenous anti-inflammatory negative feedback regulatory role (180, 181). Itaconate is generated from the TCA cycle intermediate cis-aconitate by aconitate decarboxylase 1 (ACOD1), encoded by immune responsive gene 1 (IRG1), and rapidly accumulates in macrophages upon stimulation with lipopolysaccharide (LPS) or similar agents (182, 183). Mechanistically, itaconate alkylates Kelch-like ECH-associated protein 1 (Keap1), thereby activating the Nrf2 pathway, inducing antioxidant and anti-inflammatory gene expression, and reducing the production of ROS and pro-inflammatory cytokines (184, 185). Additionally, itaconate inhibits SDH, partially blocking succinate oxidation to fumarate in early inflammation, which further elevates succinate levels (182). This effect results in the accumulation of succinic acid; however, the levels of IL-1β and IL-6 secreted by macrophages are significantly reduced, as aconitic acid simultaneously activates Nrf2 and inhibits the NF-κB and NLRP3 pro-inflammatory pathways (186). Evidence from animal studies supports the protective role of itaconate; administration of the cell-permeable derivative 4-octyl itaconate (4-OI) in ischemia–reperfusion and other inflammatory injury models markedly attenuated acute myocardial and hepatic damage, suppressed macrophage-derived inflammatory cytokines, and promoted angiogenesis and functional recovery in injured tissues (185, 187). Conversely, IRG1-deficient mice have exhibited more prolonged and intense inflammatory responses (185). Overall, succinate and itaconate serve as the “accelerator” and “brake,” respectively, in the regulation of macrophage polarization; the former amplifies inflammation and drives M1 polarization via HIF-1α stabilization and SUCNR1 activation, whereas the latter restrains inflammatory intensity through Nrf2-mediated anti-inflammatory pathways and facilitates M1-to-M2 phenotypic transition (184). In pathological conditions such as IRI, dysregulation of these two metabolites contributes to macrophage dysfunction and polarization imbalance (182).

#### 3.3.2 Fatty acid metabolism and other metabolisms

In contrast to M1 macrophages, which preferentially rely on glycolysis for rapid energy provision, M2 macrophages primarily depend on mitochondrial oxidative phosphorylation, with FAO representing a key metabolic pathway (188, 189). Under stimulation by anti-inflammatory signals such as IL-4/IL-13, transcription factors (e.g., PPAR-γ and PPAR-σ) are activated, upregulating the expression of enzymes involved in lipid uptake and catabolism. These effects enhance fatty acid uptake and oxidation to meet the metabolic demands of the anti-inflammatory and pro-reparative M2 phenotype (189, 190). Acetyl-CoA generated from FAO enters the TCA cycle and undergoes complete oxidation, consuming large amounts of reduced nicotinamide adenine dinucleotide (NADH) via oxidative phosphorylation and elevating the intracellular NAD^+^/NADH ratio (165). Increased NAD^+^ levels are particularly critical for epigenetic regulation in macrophages. NAD^+^ serves as an essential co-factor for class III histone deacetylases (HDACs; sirtuins), including silent information regulator 1 (SIRT1) and related members, which remove acetyl groups from histones and transcription factors to modulate gene expression (191). In M2 macrophages, elevated NAD^+^ activates nuclear SIRT1 and other deacetylases, reducing histone acetylation at pro-inflammatory gene promoters, thereby suppressing their transcriptional activity. Concurrently, SIRT1 directly deacetylates inflammation-associated transcription factors, attenuating the expression of pro-inflammatory genes (192, 193). Conversely, in M1 macrophages, the Warburg effect-driven glycolysis and inhibition of mitochondrial respiration by NO and other products impair NADH reoxidation, lowering the NAD^+^/NADH ratio and compromising SIRT1 activity (194, 195). Consequently, histones and factors such as NF-κB remain highly acetylated, sustaining active transcription of pro-inflammatory genes and perpetuating the M1 inflammatory program (167). Experimental evidence demonstrated that activation of the metabolic sensor AMPK enhances FAO and mitochondrial function, increases SIRT1 activity, reprograms macrophages from the M1 to M2 phenotype, markedly reduces pro-inflammatory cytokine release, and augments the production of anti-inflammatory cytokines (196, 197). Similarly, the elevation of intracellular NAD^+^ levels suppressed macrophage inflammatory responses in various models. This effect was closely linked to enhanced deacetylase activity and pro-inflammatory gene silencing (198). Recent studies further revealed that upstream regulators, such as ubiquitin-specific protease 14 (USP14), stabilize SIRT1 protein in macrophages, reinforcing FAO and promoting M2 polarization. This finding underscores the role of the “metabolism–epigenetics” axis in macrophage fate determination. The metabolic status exerts long-term control over gene expression profiles through modulation of epigenetic enzyme activity (199).

**Figure 4:**
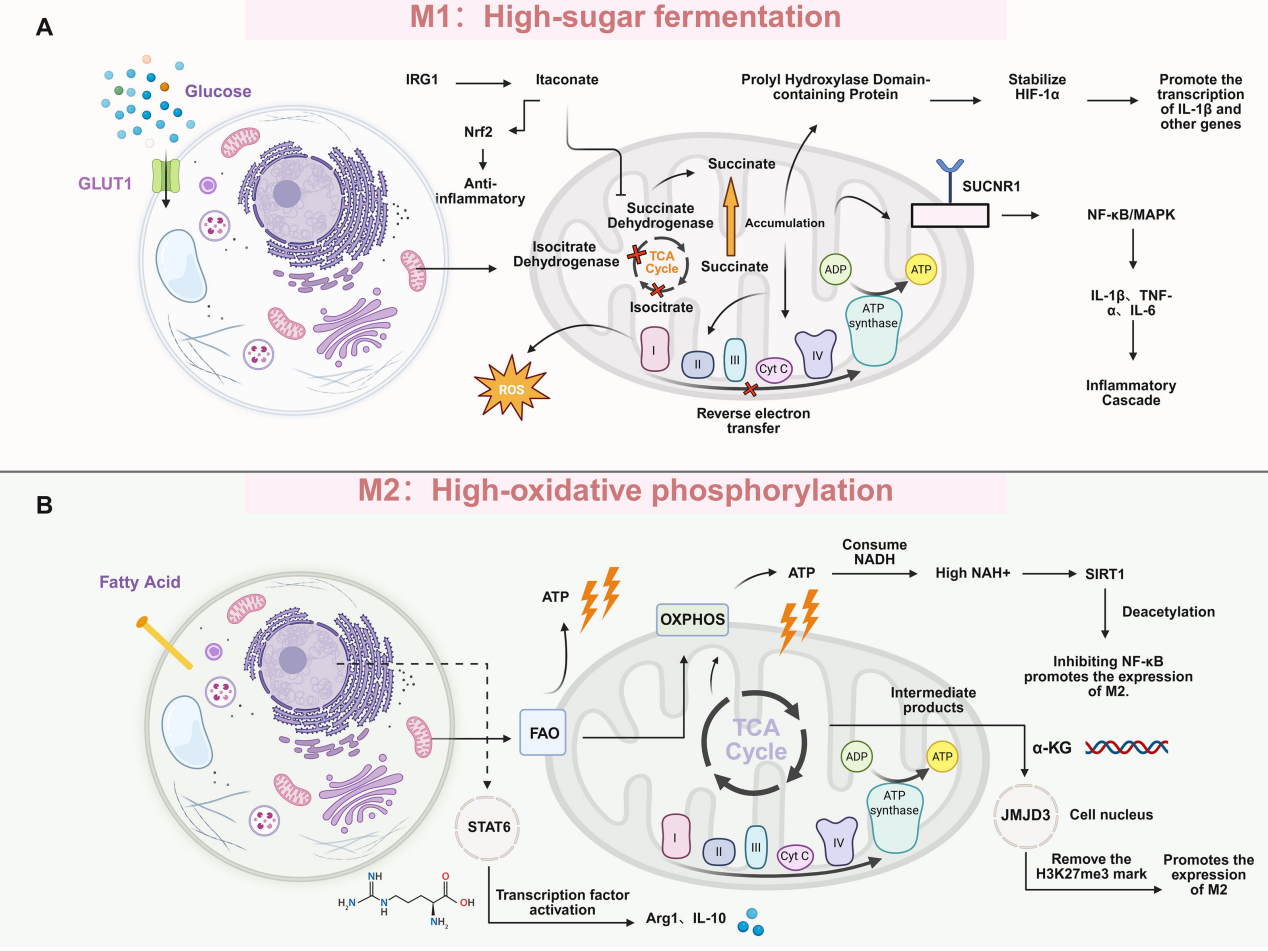
Comparative overview of M1/M2 metabolic pathways and signaling regulatory networks in IRI. **(A)** M1 macrophages exhibit a metabolic characteristic of “high glycolysis.” Following the entry of glucose into the cell through GLUT1, the TCA cycle is inhibited, resulting in the accumulation of succinic acid. Activation of the NF-κB/MAPK pathway by SUCNR1 and combination with processes such as IRG1–arachidonic acid, ROS, and HIF-1α stabilization ultimately trigger an inflammatory cascade related to pro-inflammatory factors. **(B)** M2 macrophages exhibit a metabolic characteristic of “high oxidative phosphorylation.” They utilize fatty acids for energy supply through FAO. Through the TCA cycle + OXPHOS, a large amount of ATP is generated. The M2 phenotype is promoted by activating M2 molecules such as Arg1/IL-10 through STAT6, as well as through the epigenetic regulation of SIRT1 and JMJD3. IRI, ischemia–reperfusion injury; FAO, fatty acid oxidation; OXPHOS, oxidative phosphorylation; TCA, tricarboxylic acid.

Beyond lipid metabolism, intermediates from other pathways also influence macrophage polarization via epigenetic mechanisms (200). A representative example is glutamine metabolism and its product α-ketoglutarate (α-KG) (201). Enhanced glutaminolysis in M2 macrophages increases α-KG supply to the TCA cycle, favoring the activation of α-KG-dependent histone demethylase Jumonji domain-containing protein 3 (JMJD3). This effect removes repressive histone H3 lysine 27 trimethylation (H3K27me3) marks from M2-associated gene promoters, thereby epigenetically relieving transcriptional repression and facilitating the establishment of the anti-inflammatory phenotype (201). In contrast, accumulated succinate, fumarate, and 2-hydroxyglutarate in M1 macrophages competitively inhibit α-KG-dependent demethylases, impeding the epigenetic activation of M2 genes (167). This differential metabolite availability exerts a “double-edged sword” effect on polarization fate. Specifically, sufficient α-KG biases macrophages toward M2 conversion, whereas excess succinate/fumarate prolongs the open chromatin state of pro-inflammatory genes, anchoring cells in the M1 functional state. Thus, oxidative metabolism supplies energy and shapes macrophage functional phenotypes by altering intracellular NAD^+^ levels and metabolite concentrations, thereby regulating histone modifications and transcription factor activity (202). In pathological conditions such as IRI, promoting enhanced FAO and mitochondrial biogenesis in macrophages holds promise for augmenting their anti-inflammatory/pro-reparative functions, preventing sustained M1 bias due to metabolism–epigenetics dysregulation, and restoring the balance between inflammation and repair (**Figure 4**).

### 3.4 Epigenetic regulation

Dynamic epigenetic modifications are essential for stabilizing macrophage phenotypes, and IRI-induced epigenetic dysregulation represents a key driver of M1/M2 imbalance (20). Firstly, aberrant histone modifications are common in IRI. Under physiological conditions, activating histone H3 lysine 4 trimethylation (H3K4me3) marks at M1-associated gene promoters progressively diminish as inflammation resolves, preventing sustained pro-inflammatory transcription. However, persistent danger signals in ongoing injury abnormally enhance histone methyltransferase activity. This maintains high H3K4me3 levels and keeps pro-inflammatory genes in a chronically “open” state, thereby amplifying tissue damage (203, 204). Conversely, repressive H3K27me3 marks at key M2 gene promoters accumulate abnormally due to reduced demethylase activity in IRI, directly suppressing anti-inflammatory and reparative pathways and impairing M2 function (204).

Additionally, exosome-mediated transfer of microRNAs (miRNAs) plays a significant role in the intercellular “reprogramming” of macrophage phenotypes. Studies have shown that pro-M1 miRNA (miR-155) is highly expressed during IRI and enhances NF-κB signaling by suppressing negative regulators such as SOCS1, thereby maintain the M1 polarization level (205, 206). Li et al. directly confirmed that miR-155 deficiency inhibits the excessive activation of hepatic Kupffer cells (KCs), promotes their shift toward the M2 phenotype, reduces TNF-α and IL-6 production while increasing IL-10 expression, and significantly alleviates liver IRI injury through phenotype modulation of KCs (207). Similarly, Ding et al. found that renal tubular epithelial cells release exosomal miR-374b-5p following hypoxic injury in renal IRI. Upon uptake by macrophages, this miRNA directly targets and suppresses Socs1, driving polarization toward the pro-inflammatory M1 phenotype and exacerbating renal inflammation and tissue damage. This evidence highlights the “exosomal miR-374b-5p–Socs1–M1 polarization” axis as an intervenable pathological pathway (208).

**Figure 5:**
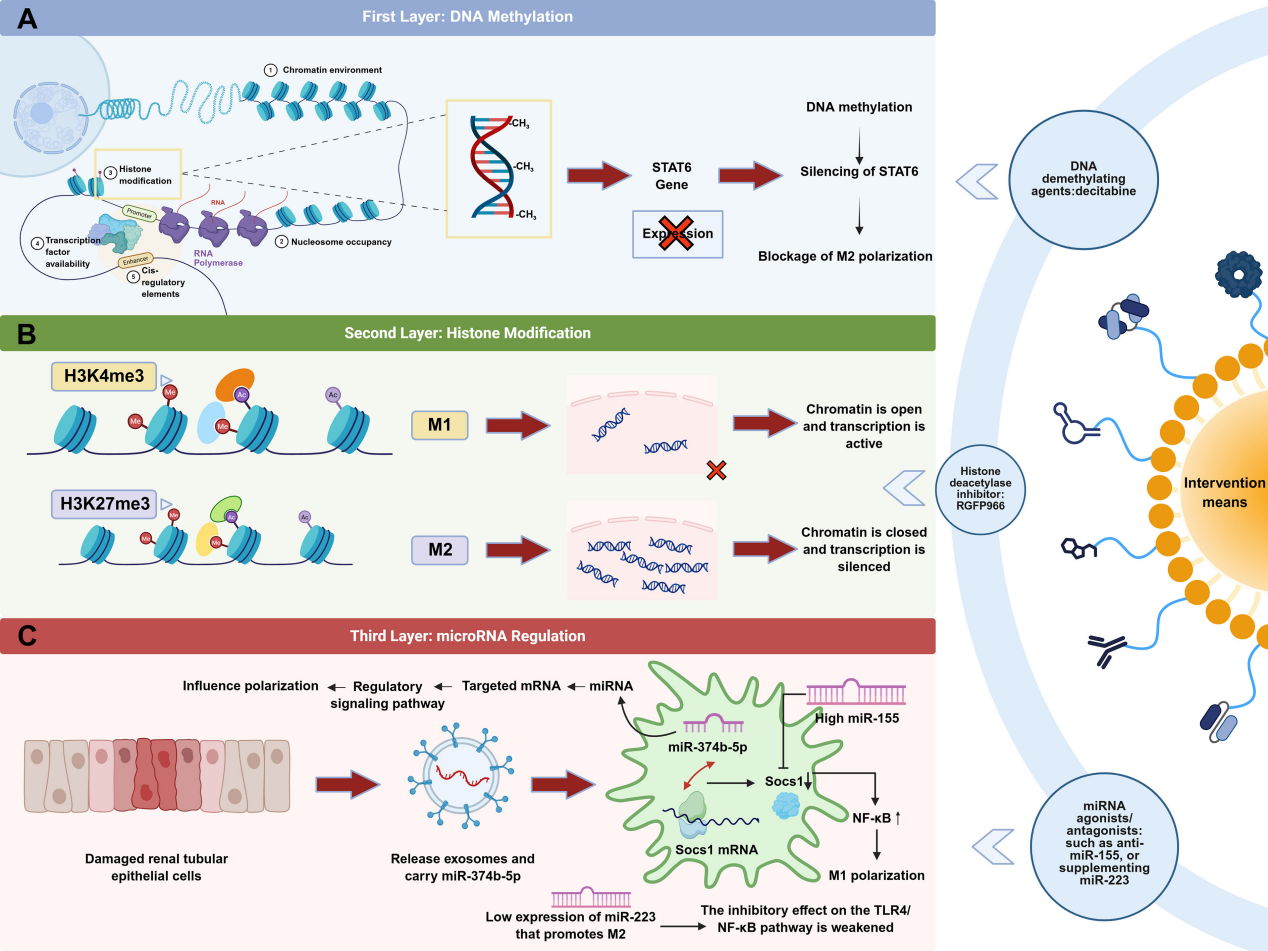
Schematic representation of epigenetic regulation mediating macrophage polarization. **(A)** The DNA methylation of the STAT6 gene silences it and blocks M2 polarization, as demethylating agents (e.g., decitabine) can be used for intervention. **(B)** M1 corresponds to the H3K4me3 modification that opens the chromatin and activates transcription, while M2 corresponds to the H3K27me3 modification that closes the chromatin and silences transcription. Histone deacetylase inhibitors, such as RGFP966, can be used for intervention. **(C)** Damaged renal tubular epithelial cells release exosomes containing miR-374b-5p, which in combination with the high expression of miR-155 and the low expression of miR-223 weaken the inhibition of the TLR4/NF-κB pathway and promote M1 polarization.

In contrast, pro-M2 miRNAs (e.g., miR-223) are significantly downregulated during renal IRI (209). Under normal conditions, these miRNAs maintain an anti-inflammatory differentiation bias in macrophages and balance local inflammation by inhibiting TLR4 and NF-κB signaling (210, 211). In IRI, however, their reduction abolishes restraint on M1 activation pathways, hindering the upregulation of anti-inflammatory cytokines such as IL-10 and further increasing TNF-α and IL-1β release. Ultimately, these effects lead to the formation of a bidirectional imbalance network characterized by upregulation of pro-M1 miRNAs and downregulation of pro-M2 miRNAs (205, 208). Thus, multilayered epigenetic aberrations—from histone modifications and DNA methylation to exosomal miRNAs—collectively establish and maintain M1 dominance while weakening M2 function, thereby preventing timely transition from inflammation to repair. This phenomenon also offers therapeutic opportunities for IRI. For example, epigenetic drugs can be employed to correct aberrant H3K4me3/H3K27me3 and DNA methylation marks. Furthermore, strategies involving exosomes or oligonucleotides can inhibit miR-155 and miR-374b-5p, while restoring the levels of pro-M2 miRNAs (e.g., miR-223). Such approaches aim to reestablish the M1/M2 balance at the epigenetic level, ameliorate inflammatory injury in IRI, and enhance the quality of tissue repair (**Figure 5**).

### 3.5 Organ-specific microenvironmental differences

The anatomical structures, physiological functions, energy metabolism characteristics, and body fluid compositions of different organs exhibit inherent differences. These factors collectively determine that the manifestations of macrophage polarization imbalance in IRI also vary across organs, displaying distinct organ-specific features. In the high-oxygen-consuming heart, large amounts of DAMPs, such as myoglobin released following cardiomyocyte necrosis, preferentially activate the TLR4 signaling pathway in cardiac macrophages, thus rapidly amplifying local pro-inflammatory signals (212). Simultaneously, matrix stiffening and the fibrotic microenvironment in the infarct zone sustain the secretion of CCL2 by M1 macrophages, continuously recruiting CCR^2+^ monocytes/macrophages to the injured area. This process establishes a vicious cycle characterized by “persistent inflammation-driven–fibrosis reinforcement–re-amplified inflammation” (213). This phenomenon is closely associated with the high expression of TLR4 and CCR2 in cardiac macrophages, resulting in prolonged M1 dominance and delayed M2-mediated anti-inflammatory and tissue remodeling processes, ultimately leading to organ dysfunction.

The specificity of brain tissue lies in the selective permeability of the blood–brain barrier (BBB) and its dense glial network structure (214). Following cerebral IRI, BBB damage allows peripheral monocytes/macrophages to infiltrate the central nervous system parenchyma and closely interact with resident microglia (215). Large amounts of ATP released by neurons and glial cells act on P2X7 receptors, driving microglia toward an M1 phenotype and releasing neurotoxic factors such as NO and IL-1β, which further damage neurons and cerebral endothelial cells (216). Concurrently, persistent local hypoxia upregulates HIF-1α levels, thereby suppressing M2-related gene expression and impeding repair processes (217). In summary, the phenotypic cross-regulation and dynamic switching between peripheral macrophages and resident microglia profoundly participate in the entire pathological process of cerebral IRI, from initiation to progression, and have become the core regulatory hub determining post-ischemic neurological prognosis.

The liver, receiving blood supply from both the portal vein and hepatic artery, exhibits a unique “gut–liver axis” effect (218). The portal vein continuously delivers abundant gut-derived metabolites and microbial components that persistently shape the receptor expression profile of KCs, establishing them as the primary effector cells in the gut–liver axis functional regulation (219, 220). During hepatic IRI, damage to the intestinal mucosal barrier facilitates the translocation of LPS into the bloodstream. These gut-derived toxins continuously drive KCs toward M1 polarization and maintain phenotypic stability through specific activation of the TLR4/NF-κB signaling pathway on the surface of KCs (221, 222). Moreover, hepatocyte injury and post-ischemic cholestasis lead to bile acid accumulation in the local microenvironment, further inhibiting M2-associated anti-inflammatory metabolic pathways and the catalytic activity of Arg1. These effects significantly impair endogenous anti-inflammatory responses and the capacity for tissue repair in the liver (223, 224). The aforementioned factors cause temporal overlap and spatial mutual reinforcement between the pro-inflammatory drive and repair suppression, rendering the liver prone to a persistent “high inflammation–low repair” state.

Renal IRI is closely linked to the “tubule–interstitium” axis and the hyperosmotic medullary microenvironment (225). After renal IRI, damaged tubular epithelial cells release pleiotropic stress molecules and other DAMPs, thus activating pro-inflammatory signaling pathways in renal interstitial macrophages and inducing M1 polarization (226). Additionally, the combination of the intrinsically hyperosmotic medullary environment with intense oxidative stress during reperfusion suppresses the mitochondrial oxidative metabolism and fatty acid oxidation pathways required for M2 polarization, thereby reducing the synthesis and secretion of anti-inflammatory cytokines such as IL-10. Under this dual imbalance of the metabolic microenvironment and signaling regulation, the kidney is more susceptible to unresolvable inflammation and defective tissue repair, markedly increasing the risk of renal fibrosis and irreversible functional damage (227).

**Figure 6:**
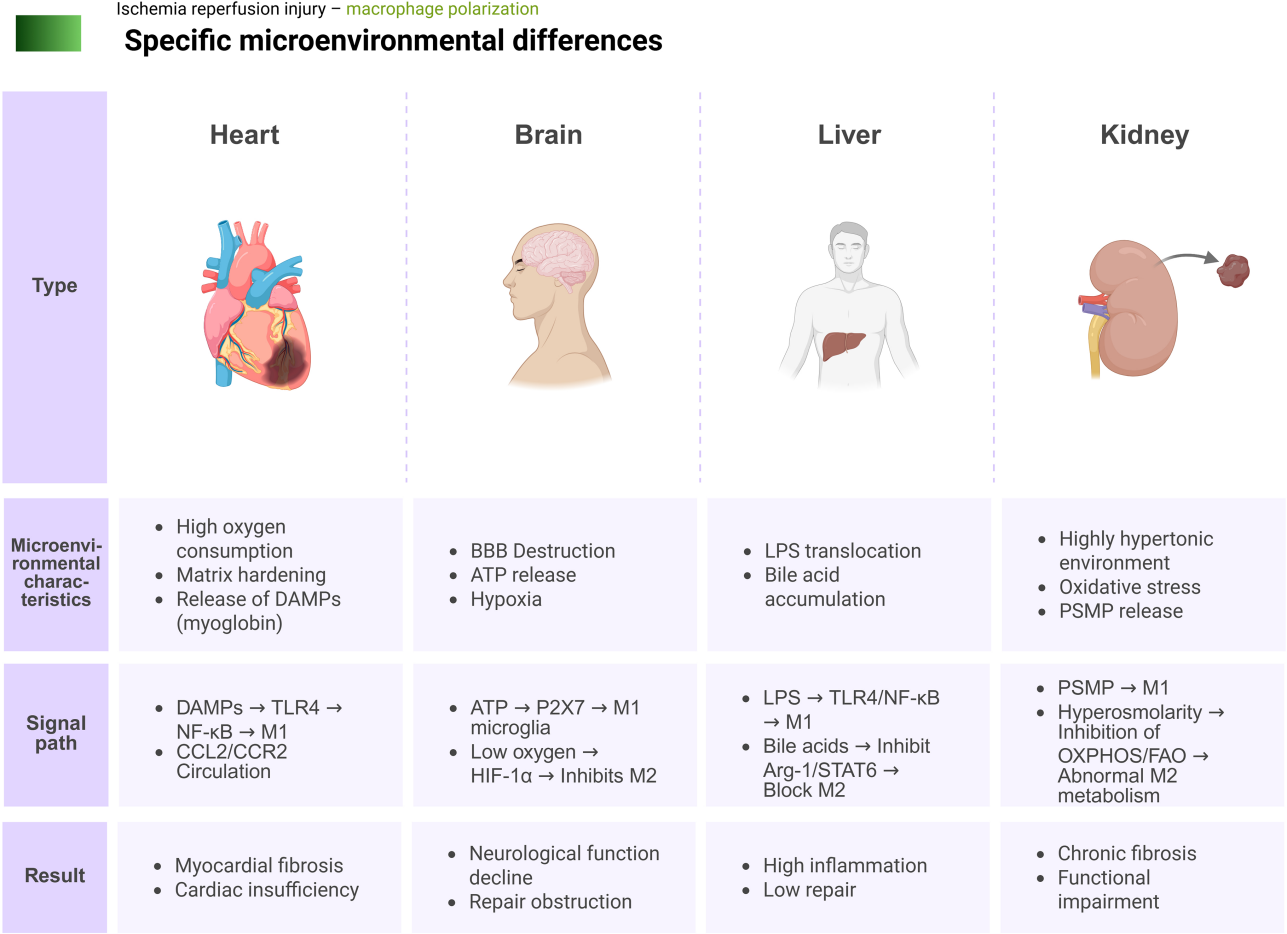
Comparison of the specificity of four common organs in IRI. BBB, blood–brain barrier; DAMPs, damage-associated molecular patterns; FAO, fatty acid oxidation; IRI, ischemia–reperfusion injury; LPS, lipopolysaccharide; OXPHOS, oxidative phosphorylation; PSMP, pleiotropic stress molecule.

Thus, the inherent heterogeneity in anatomical structure, barrier morphology, and metabolic background among typical IRI target organs (i.e., heart, brain, liver, and kidney) shapes the landscape of macrophage polarization imbalance. From a common perspective, these organs universally exhibit high DAMP burden in the acute phase, excessive activation of pro-inflammatory pathways (e.g., TLR/NF-κB and NLRP3), and sustained amplification of chemotactic axes (e.g., CCL2/CCR2), resulting in varying degrees of prolonged M1 dominance. In contrast, M2 anti-inflammatory and repair metabolic programs based on OXPHOS/FAO, along with signaling pathways such as STAT6/PPAR-γ, are suppressed under conditions of high oxidative stress, matrix stiffening, or accumulation of harmful metabolites, forming an overall pattern of “enhanced pro-inflammatory drive-blocked repair programs.” Nevertheless, each organ displays critical differences in regulatory nodes. For instance, the heart tends to prolong the M1 temporal window due to matrix stiffening and persistent CCR^2+^ monocyte infiltration (89). The brain is dominated by interactions between peripheral monocytes/macrophages and microglia following BBB disruption, forming neurotoxic inflammation sustained by the P2X7–HIF-1α axis (224). The liver, reliant on LPS and bile acid signals from the gut–liver axis input, pushes KCs into a highly TLR4-sensitive and difficult-to-reverse M1-biased phenotype (218). The kidney, under superimposed stress of hyperosmolarity and reperfusion oxidative stress, exhibits long-term restriction of M2-dependent mitochondrial metabolism and fatty acid oxidation, making it more prone to transition from subacute inflammation to chronic fibrosis (227). This organ-specific heterogeneity perspective suggests that future macrophage-targeted interventions must simultaneously address “common axes” and “organ-specific axes.” M1/M2 balance can be truly restored at the systemic level only by precisely defining the optimal temporal windows and combination targets in different organs. This approach could provide core theoretical support for personalized and precision intervention in IRI (**Figure 6**).

## 4 Mechanical regulation of macrophage polarization in IRI

As highly plastic immune cells, macrophages undergo phenotypic polarization in response to various physical and mechanical signals. This process is regulated by biochemical factors. Hence, macrophages exhibit distinct functional states in different microenvironments. Recent studies have demonstrated that in the context of IRI, mechanical signals such as shear stress and matrix stiffness in organs with specific biomechanical properties (heart and kidney), activate mechanosensitive receptors on the macrophage surface, including Piezo type mechanosensitive ion channel 1 (Piezo1) and transient receptor potential vanilloid subtype 4 (TRPV4). This activation triggers downstream signaling axes involving Yes-associated protein/transcriptional co-activator with PDZ-binding motif (YAP/TAZ) nuclear translocation and calcium-dependent nuclear factor of activated T cells (NFAT), thereby directly modulating the direction of macrophage polarization (24, 228). More importantly, this mechanosensing process involves extensive crosstalk with cellular metabolic reprogramming and epigenetic states, forming a sophisticated regulatory network (229).

### 4.1 Shear stress and polarization

Blood flow shear stress constitutes a critical biomechanical factor in the circulatory system. Studies have demonstrated that the magnitude and pattern of shear stress influence the phenotype of intravascular macrophages and monocytes (230–232). For instance, in atherosclerosis models, distinct regional blood flow patterns induce differential macrophage polarization. In areas of low or disturbed shear stress, macrophages preferentially adopt an M1 phenotype, exhibiting elevated pro-inflammatory cytokine expression and exacerbating lesion progression. In contrast, within regions exposed to oscillatory shear stress, local macrophages display partial M2-like anti-inflammatory characteristics (231, 233). *In vitro* investigations employing multiaxial mechanical loading models have further revealed that pure shear or compressive stress alone upregulates pro-inflammatory gene expression in monocytes, whereas combined shear-compression stimuli induce the production of certain anti-inflammatory cytokines. Thus, shear stress modulates the pro-inflammatory/anti-inflammatory cytokine expression ratio in macrophages by altering their perception of the mechanical microenvironment, thereby exerting regulatory effects on inflammatory responses and tissue remodeling (234, 235). This mechanostimulatory effect is likely mediated, at least in part, by downstream signaling triggered when ion channels and cytoskeletal linker proteins on the macrophage membrane sense fluid friction forces (236, 237). Unlike traditional biochemical stimuli such as TLR activation, the influence of shear stress provides a novel perspective for understanding the pathogenesis of IRI.

### 4.2 Matrix stiffness and polarization

The mechanical stiffness of tissue or extracellular matrix represents another critical physical parameter in the macrophage microenvironment (24). During tissue injury or fibrosis progression, matrix stiffness frequently undergoes alterations that influence macrophage function via mechanosensing pathways (238). *In vitro* culture systems and animal models have confirmed that increased substrate stiffness can profoundly alter macrophage polarization states (239). For example, when bone marrow-derived macrophages are cultured on hydrogels of varying stiffness, soft substrates (1 kPa) preferentially promote an M2 anti-inflammatory phenotype, whereas stiff substrates (50 kPa) drive polarization toward a pro-inflammatory M1 state (239). In murine skin fibrosis models, pathological regions exhibit markedly elevated matrix stiffness accompanied by increased M1 macrophage infiltration; genetic ablation of the mechanosensitive channel TRPV4 significantly attenuates stiffness-induced M1 polarization (238). TRPV4 serves as a key ion channel through which macrophages sense matrix rigidification; its knockout abolishes stiffness-dependent upregulation of pro-inflammatory genes, whereas TRPV4 overexpression restores the M1 phenotype under high-stiffness conditions (238). Similarly, the mechanosensitive channel Piezo1 plays a central role in macrophage mechanotransduction (24). Stiff matrices upregulate Piezo1 expression and trigger Piezo1-mediated Ca^2+^ influx; the resulting cytosolic Ca^2+^ surge activates calcineurin, which dephosphorylates YAP, releasing it from inhibitory cytoplasmic complexes and promoting its nuclear translocation. Subsequently, nuclear YAP forms complexes with TEA domain (TEAD) family transcription factors, binds to promoter regions of M1-associated genes, directly drives the transcription of pro-inflammatory mediators, and simultaneously represses M2-related gene activation, thereby skewing macrophages toward an M1 phenotype (239–241). Collectively, these findings demonstrate that changes in matrix stiffness exert a profound influence on macrophage fate through activation of mechanoreceptors and downstream mechanotransductive pathways. In summary, the relationship between mechanical forces and macrophage polarization is highly context-dependent. In infectious or fibrotic inflammatory settings, stiff matrices typically drive pro-inflammatory M1 responses. However, in certain contexts such as the tumor microenvironment, mechanical cues may induce immunosuppressive M2-like states via alternative pathways. This context-dependent characteristic suggests that therapeutic applications leveraging mechanoregulation of macrophage polarization adhere to the same principles. This opens new avenues and providing innovative strategies for mechanotherapy-centered interventions in IRI.

### 4.3 Role of mechanosensors (Piezo1/TRPV4)

Mechanoreceptors, including integrin receptors and mechanosensitive ion channels, are present on the surface of macrophages. They serve as pivotal transducers that convert physical forces into intracellular biochemical signals and constitute essential mediators of mechanoregulated macrophage polarization (242, 243). Integrins link the cytoskeleton to the extracellular matrix. Upon mechanical loading, they undergo clustering and activate downstream signaling molecules, such as focal adhesion kinase (FAK) and MAPK pathways, thereby modulating macrophage migration, phagocytosis, and polarization. Studies have shown that cyclic stretch markedly upregulates integrin alpha M (CD11b) expression in macrophages, while downregulating Piezo1 channel levels. Knockdown of either CD11b or Piezo1 abolishes the effects of mechanical stretch on macrophage activation (242). Notably, crosstalk exists between integrin CD11b and Piezo1. Silencing one triggers compensatory upregulation of the other, indicating that integrin-mediated adhesion signaling and ion channel-dependent mechanosensing coordinately determine the intensity of downstream inflammatory responses in macrophages (242). Mechanical stretch relies on dynamic actin cytoskeleton remodeling. Inhibition of actin polymerization blocks the regulatory effects of mechanical stress on macrophage phenotype, demonstrating that mechanical signals must be transmitted through the cytoskeleton to signaling complexes to influence transcriptional programs (244, 245). Within mechanosensitive pathways, YAP/TAZ (canonical mechanotransductive transcriptional co-activators) have recently been implicated in macrophage phenotypic regulation. High tension and stiff substrates promote YAP/TAZ nuclear translocation in macrophages, where they cooperate with TEAD and other transcription factors to regulate downstream gene expression (246–248). For instance, Piezo1 activation, as described above, facilitates YAP nuclear localization and induces M1-associated genes (239). Importantly, YAP/TAZ respond to matrix stiffness, shear stress, and cellular deformation, and may be involved in macrophage polarization under diverse mechanical stimuli (249). Furthermore, mechanosensitive ion channels such as TRPV4 mediate calcium signaling to activate pathways (e.g., AKT and NF-κB), thereby altering macrophage activation states (238). In summary, macrophages integrate physical cues into intracellular signaling networks through a repertoire of mechanoreceptors, enabling precise regulation of their functional fate.

### 4.4 Crosstalk regulation involving mechanotransduction, metabolism, and epigenetics

The regulation of macrophage polarization by mechanical stimuli is inextricably linked to the coordinated involvement of cellular metabolic pathways and epigenetic mechanisms (250, 251). Metabolically, pro-inflammatory M1 macrophages primarily rely on glycolysis for energy supply, whereas M2 macrophages predominantly utilize oxidative phosphorylation and fatty acid oxidation (169, 252). This metabolic dichotomy provides critical targets for mechanosignaling modulation. Mechanical cues can alter macrophage metabolic reprogramming by influencing the activity of key transcription factors and co-factors (253). For instance, mechanical stretch and matrix stiffening stabilize and activate HIF-1α in macrophages, thereby enhancing glucose uptake and lactate production and reinforcing M1 metabolic profiles (253, 254). The mechanotransductive effector YAP is also directly linked to cellular metabolism. YAP forms complexes with TEAD transcription factors and binds to promoter regions of metabolic enzyme genes to regulate their transcription (255). The YAP-TEAD complex directly modulates genes involved in metabolic substrate utilization, including those encoding tricarboxylic acid cycle enzymes (e.g., IDH2), thereby exerting a central role in the transcriptional control of metabolic enzymes (256). Studies have shown that under hypoxic mechanical stress, YAP directly binds the promoter of 6-phosphofructo-2-kinase/fructose-2,6-bisphosphatase 3 (PFKFB3) and enhances its expression, resulting in increased glycolytic flux (257, 258). Thus, it can be inferred that in macrophages, YAP-mediated mechanical signaling may promote glycolysis through upregulation of enzymes such as PFKFB3, thereby biasing polarization toward the M1 phenotype. Another consequence of mechanical stimulation is the generation of calcium transients and second messenger changes. Piezo1 and TRPV4-mediated Ca^2+^ influx activates the phosphatase calcineurin, which dephosphorylates and promotes the nuclear translocation of NFAT transcription factors, leading to upregulation of pro-inflammatory genes and metabolic regulators (259, 260).

Concurrently, the mechanical microenvironment can physically alter the nuclear architecture and chromatin organization, thereby inducing epigenetic reprogramming. High-stiffness substrates or tensile forces may modify nuclear envelope tension and chromatin accessibility in macrophages, influencing the recruitment of histone-modifying enzymes and DNA methylation patterns. Reports indicate that cyclic stretch increases chromatin openness at certain inflammatory gene loci, facilitating transcription factor binding and promoting pro-inflammatory gene expression while suppressing anti-inflammatory gene transcription (261, 262).

**Figure 7:**
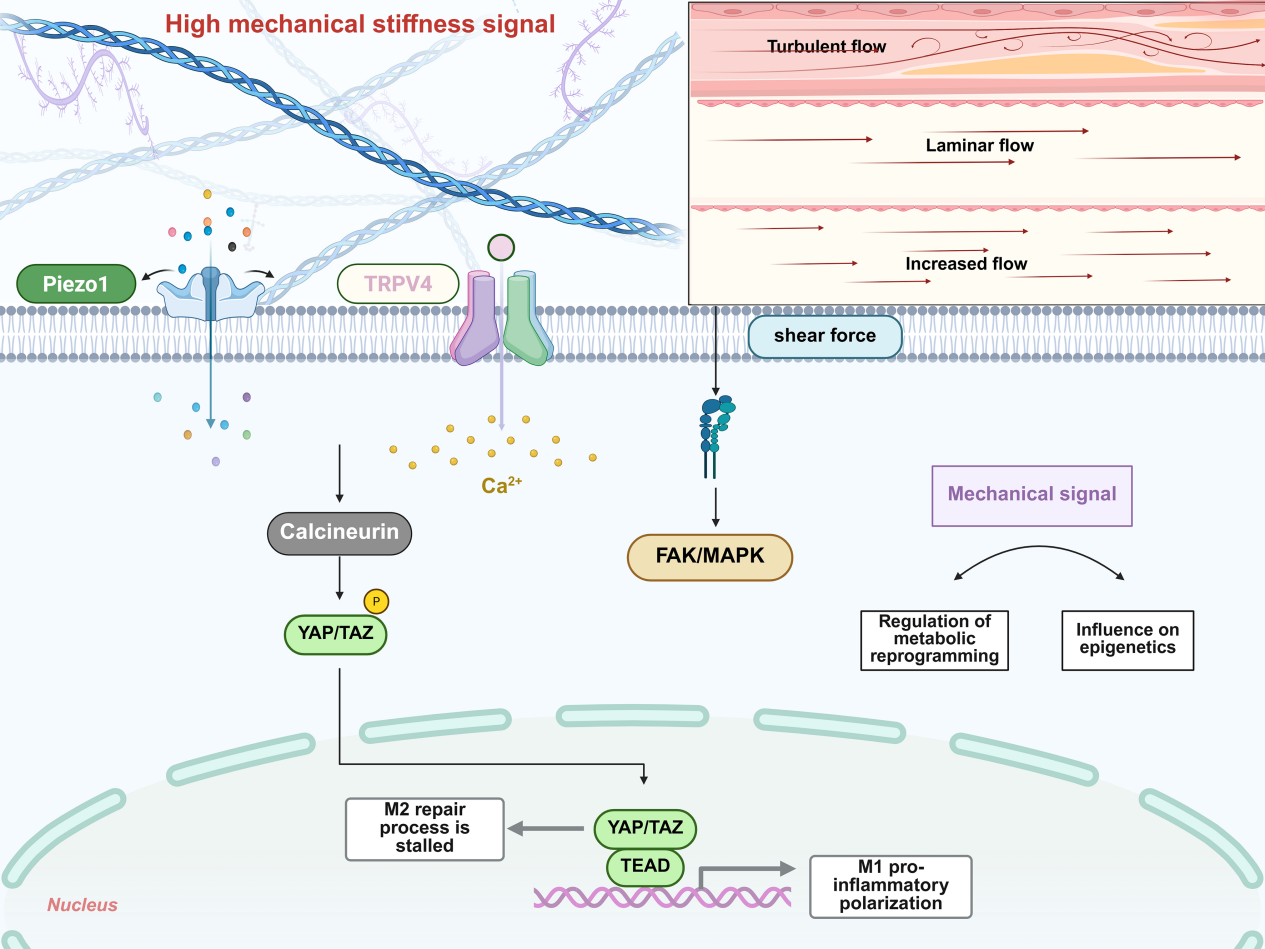
Diagram of the molecular mechanisms underlying mechanical signal stimulation in IRI. The high mechanical hardness signal is transmitted through Piezo1 and TRPV4, which activate the calcineurin–YAP/TAZ pathway and the Ca^²+^ signal, respectively. Blood flow shear force (including forms such as turbulence, laminar flow, and increased flow rate) activates the FAK/MAPK pathway. Ultimately, YAP/TAZ binds to TEAD, blocking the M2 repair process and promoting the M1 pro-inflammatory polarization, while mechanical signals can also regulate metabolic reprogramming and epigenetics. IRI, ischemia–reperfusion injury.

Mechanical signaling is also profoundly shaped by organ-specific heterogeneity, as manifested in the cyclic stretch induced by repeated myocardial contraction coupled with abrupt stiffness increases in infarcted regions (263–265), the low-stiffness neural matrix and pulsatile shear forces in brain tissue (266, 267), the low-shear sinusoidal environment and portal pressure fluctuations in the liver (268, 269), and the unique flow velocity gradients and osmotic differences at the corticomedullary junction in the kidney (270). These distinct biomechanical milieus effectively preset the sensitivity thresholds and response patterns of mechanosensing apparatuses such as Piezo1, TRPV4, and the integrin/FAK axis in each organ. The resulting mechanical inputs form intricate feedback loops with local metabolic reprogramming, cytokine/chemokine networks, and epigenetic programs mediated by histone modifications, DNA methylation, and non-coding RNAs. Aberrant mechanical loading perpetuates M1 bias by amplifying pro-inflammatory metabolism and opening pro-inflammatory chromatin domains. Moreover, persistent inflammation and metabolic dysregulation reciprocally remodel the matrix composition and stiffness, further altering the mechanomicroenvironment. Ultimately, this coupled dysregulation of mechanical, metabolic, inflammatory, and epigenetic circuits generates organ-specific trajectories of M1/M2 polarization imbalance in the heart, brain, liver, and kidney, collectively culminating in impeded inflammation resolution and aberrant repair programs (**Figure 7**).

## 5 Clinical significance and interventions for macrophage polarization imbalance in IRI

### 5.1 Small-molecule drug interventions (targeting transcription, metabolism, and epigenetics)

#### 5.1.1 Transcriptional inhibitors

Strategies centered on suppressing pro-inflammatory transcriptional signaling to block M1 macrophage activation have yielded several promising pharmacological agents in recent years. For instance, the multi-target tyrosine kinase inhibitor sunitinib can specifically inhibit the JAK2/STAT1/3 signaling pathway, thereby promoting macrophage polarization toward the anti-inflammatory M2 phenotype and alleviating inflammatory liver damage mediated by IRI (271). Similarly, in renal ischemia–reperfusion models, the flavonoid compound scutellarin inhibited key pro-inflammatory pathways in macrophages, including MAPK/activator protein-1 (MAPK/AP-1), reduced M1 polarization and inflammatory cytokine production, and significantly ameliorated tissue injury (272, 273). By downregulating the activity of critical transcription factors such as NF-κB and STAT, these transcriptional inhibitors attenuate excessive inflammatory responses during ischemia–reperfusion. This ability positions them as effective therapeutic candidates for systemically restoring macrophage phenotypic balance and improving outcomes in IRI.

#### 5.1.2 Metabolic regulators

Reshaping macrophage metabolic reprogramming to alter the polarization status represents another pivotal strategy for intervening in IRI. For example, the antidiabetic agent metformin activates the AMPK pathway and suppresses the pro-inflammatory metabolism and NLRP3 inflammasome activation. Consequently, it reduces the release of inflammatory mediators (e.g., TNF-α and IL-6) and mitigates myocardial IRI (274–276). Agonists of PPAR-γ promote macrophage polarization toward the M2 phenotype and enhance anti-inflammatory effects (277, 278). Studies have demonstrated that curcumin inhibits NF-κB-mediated inflammatory responses by activating nuclear PPAR-γ in macrophages, thereby protecting the liver from IRI-induced damage (279, 280). Furthermore, compounds targeting key metabolic coenzymes, such as the nicotinamide phosphoribosyltransferase (NAMPT) inhibitor FK866, decrease intracellular NAD levels and PARP1 activity in macrophages. These effects lead to downregulation of M1 markers (e.g., CD86 molecule, iNOS, TNF-α, and IL-1β) and an overall shift from inflammatory to reparative phenotypes, ultimately alleviating hepatic IRI-associated inflammation (281, 282). This array of metabolic modulators achieves functional phenotypic reprogramming of macrophages by altering their energy metabolism and metabolite-dependent signaling pathways, thereby contributing to the amelioration of IRI-triggered immune inflammation.

#### 5.1.3 Epigenetic modulators

As previously discussed, epigenetic mechanisms play a pivotal role in regulating macrophage polarization. Epigenetic modulators can exert long-term control over the transcriptional expression of inflammation-related genes by altering the structural stability of chromatin, thereby influencing the macrophage polarization balance. Therefore, these agents have emerged as a prominent research focus for IRI intervention. In models of ischemic injury, selective HDAC inhibitors have demonstrated pronounced anti-inflammatory effects. For instance, the HDAC3-specific inhibitor RGFP966 relieves the HDAC3-mediated transcriptional repression of anti-inflammatory genes, reduces the post-ischemic expression of M1-associated factors (e.g., TNF-α, IL-1β, and iNOS) in macrophages, promotes polarization toward the M2 anti-inflammatory phenotype, and ultimately improves neurological deficits and reduces infarct volume following cerebral IRI (283–285). Conversely, inhibitors of histone acetyltransferases (HATs) can also achieve anti-inflammatory regulation. The p300/CREB-binding protein (p300/CBP) co-activator inhibitor A-485, significantly decreases histone H3 acetylation at the promoters of pro-inflammatory genes by selectively targeting p300/CBP acetyltransferase activity. This effect suppresses the aberrant overexpression of these genes, driving macrophage polarization toward the M2 phenotype, as well as mitigating IRI-induced tissue inflammation and pathological damage (286). In summary, epigenetic drugs provide a novel perspective for the targeted modulation of macrophage polarization by precisely regulating chromatin accessibility and the epigenetic modification status of inflammatory genes. These agents have exhibited considerable potential to alleviate inflammatory injury in models of cerebral ischemia, myocardial ischemia, and related conditions.

### 5.2 Biomacromolecules and cytokine therapies

#### 5.2.1 Cytokine interventions

Macrophage phenotypic polarization is profoundly influenced by the cytokine network within the microenvironment. Therefore, targeted reversal of macrophage polarization states can be achieved through exogenous supplementation of key cytokines or activation of their receptor signaling, thereby alleviating excessive inflammatory responses triggered by IRI. The archetypal anti-inflammatory cytokine IL-10 exemplifies this approach. By engaging the IL-10 receptor (IL-10R) on macrophages, IL-10 activates the JAK1/STAT3 pathway, induces the expression of multiple anti-inflammatory genes, and suppresses pro-inflammatory signaling such as NF-κB, thereby driving polarization toward the M2 phenotype. Preclinical animal studies and translational research have demonstrated that administration of exogenous IL-10 significantly reduces the levels of pro-inflammatory cytokines in reperfused tissues, promotes local macrophage polarization to the M2 phenotype, attenuates acute injury in organs such as the kidney and heart, and improves long-term outcomes (287). Beyond classical IL-10, the recently identified IL-1 family member IL-38 exhibits comparable immunomodulatory properties. Recognized as an endogenous antagonist of the IL-36 receptor, IL-38 negatively regulates the IL-36 inflammatory axis while directly modulating the production of macrophage-associated cytokines. In myocardial IRI models, IL-38 supplementation promotes the M1-to-M2 phenotypic shift, suppresses excessive NLRP3 inflammasome activation, and enhances the secretion of anti-inflammatory cytokines such as IL-10 and TGF-β. These effects mitigate myocardial inflammatory damage through multiple mechanisms (288, 289). Notably, IL-38 can also bind IL-1 receptor accessory protein-like 1 (IL-1RAPL1) and activate the JNK/AP-1 pathway, indirectly regulating IL-6 production and conferring bidirectional immunomodulatory effects (288, 290).

Meteorin-like (Metrnl), also designated IL-41, is another cytokine of considerable interest. Initially identified in M2 macrophages and skeletal muscle, Metrnl modulates energy metabolism and inflammation via paracrine mechanisms (291). In myocardial and hepatic IRI models, elevation of Metrnl levels through recombinant protein administration or gene vector-mediated overexpression significantly reduced infarct volume and ischemic-zone apoptosis (196, 292). Mechanistic studies confirmed that Metrnl specifically activates the AMPK pathway in macrophages, thus facilitating M1-to-M2 polarization, decreasing the expression of pro-inflammatory mediators (e.g., IL-1β and TNF-α), and increasing IL-10 production. Moreover, Metrnl-conditioned anti-inflammatory macrophages secrete protective factors, including VEGF, which exert direct paracrine protection on reperfused cardiomyocytes (196). These effects are attenuated upon AMPK signaling blockade, underscoring that Metrnl primarily reprograms macrophage functional phenotype through metabolic sensing pathways. In summary, cytokine-based interventions involving IL-10, IL-38, Metrnl, and related molecules offer diverse strategies for the targeted regulation of macrophage polarization by directly supplying exogenous anti-inflammatory signals or by relieving endogenous pro-inflammatory pathway overactivation. Ultimately, these processes confer tissue protection in IRI, attenuating excessive inflammation and establishing a favorable immune microenvironment conducive to tissue repair.

#### 5.2.2 Other biomacromolecules

Besides directly supplementing cytokines, biologic macromolecule therapies targeting macrophage surface receptors or inflammatory mediators have also demonstrated potential in the prevention and treatment of IRI. These immune intervention approaches include monoclonal antibodies, soluble receptor antagonists, and recombinant fusion proteins, which indirectly guide macrophages toward a repair-favorable phenotype by blocking key pro-inflammatory pathways or inducing competitive inhibition. Among these, monoclonal antibodies can precisely target macrophage-associated receptors or ligands. For instance, monoclonal antibodies blocking the chemokine receptor CCR2 can specifically reduce the recruitment of Ly6C^+^ inflammatory monocytes to ischemic injured tissues, thereby decreasing the local accumulation of M1 macrophages at the source, suppressing the early pro-inflammatory cascade, and mitigating acute inflammatory damage in the ischemic area (293). Antagonistic antibodies or small-molecule inhibitors targeting the macrophage TLR4 receptor (e.g., TAK-242) can inhibit DAMPs-mediated TLR4/MyD88/IRAKs/TRAF6 signaling, and reduce the activation of pro-inflammatory transcription factors (e.g., NF-κB), thereby limiting the M1 polarization tendency of macrophages (294). Another strategy involves the neutralization of key inflammatory mediators. HMGB1, a DAMP molecule released in large quantities during the early phase of IRI, drives pro-inflammatory responses in macrophages via TLR4 and the receptor for advanced glycation end products (RAGE). In IRI models, the application of anti-HMGB1 neutralizing antibodies significantly reduced inflammatory damage. For example, in a rabbit spinal cord IRI model, treatment with this antibody markedly improved spinal cord motor function scores, reduced infarct area in spinal cord tissue, and increased the neuronal survival rates. Furthermore, it inhibited the excessive activation of macrophages/microglia and neutrophil infiltration, and decreased the production of inflammatory mediators such as TNF-α, IL-6, and ROS (295). These findings underscore the value of blocking DAMPs-receptor interactions in alleviating the pro-inflammatory activity of macrophages.

Similarly, soluble receptor antagonists can achieve comparable anti-inflammatory effects by acting as “molecular decoys” to sequester inflammatory factors. IL-1Ra is the most representative example, which binds with high affinity to IL-1 receptors on the surface of macrophages without triggering downstream signaling, thereby competitively blocking the pro-inflammatory stimulation of macrophages by IL-1α/β. Studies have shown that administration of recombinant IL-1Ra reduces neutrophil infiltration and apoptosis in IRI-affected myocardial and renal tissues, thereby attenuating tissue damage. This protective effect may be associated with the downregulation of pro-inflammatory factors in macrophages and a reduction in the proportion of cells with the M1 phenotype (296, 297). Likewise, soluble receptor fusion proteins for TNF-α, such as etanercept, weaken the sustained stimulation of macrophages by TNF-α through competitive binding, helping to limit the activation of M1 macrophages. Although TNF-α exhibits certain dual roles under ischemic stress, its excessive production generally prolongs the inflammatory phase; thus, moderate antagonism of TNF-α can promote the transition of macrophages toward M2 functions, thus facilitating the resolution of inflammation (298, 299). Furthermore, recombinant fusion proteins provide more flexible tools for the regulation of immune balance. For example, fusing cytokine or receptor fragments with the immunoglobulin Fc segment can extend the *in vivo* half-life of the drug, enhance targeting, and improve bioavailability. Cytotoxic T-lymphocyte-associated protein 4-immunoglobulin (CTLA-4-Ig), such as abatacept, consists of the extracellular domain of the T-cell negative co-stimulatory molecule CTLA-4 fused to the IgG1 Fc segment. The agent can specifically bind B7 molecules on the surface of macrophages and dendritic cells, blocking the T-cell activation cascade mediated by the B7-CD28 co-stimulatory pathway. This process attenuates sustained pro-inflammatory signaling loops in macrophages at the paracrine level. Traditionally, CTLA-4-Ig has been used to suppress adaptive immune responses such as transplant rejection. Nevertheless, in the context of ischemia–reperfusion, it holds promise for indirectly correcting macrophage polarization imbalance through inhibition of macrophage-T cell interactions, thereby exerting tissue-protective effects (300, 301). In summary, each of these biological macromolecule intervention measures target the key links of the pro-inflammatory pathway from different perspectives. The common results of these measures were a reduction in M1 macrophage polarization and an increase in the number of M2 macrophages, thereby alleviating the immune pathological damage of IRI and promoting tissue repair.

### 5.3 Cell therapies and nanodelivery

#### 5.3.1 Cell therapies

In recent years, cell-based therapeutic strategies have demonstrated unique advantages in targeting the regulation of macrophage polarization for the prevention and treatment of IRI. Among these, cell therapies represented by mesenchymal stem cells (MSCs) and their derived exosomes have attracted considerable attention. MSCs possess immune-privileged properties and robust paracrine functions. These characteristics enable them to mediate the polarization of local macrophages toward the M2 phenotype at damaged sites through the secretion of various bioactive factors (302, 303). Anti-inflammatory and pro-repair factors continuously released by MSCs, such as IL-10, TGF-β, prostaglandin E2 (PGE2), lipoxin A4, and tumor necrosis factor-stimulated gene 6 (TSG-6), directly act on macrophages and other immune cells. Consequently, they inhibit the production of inflammatory mediators and upregulate the expression of M2-associated genes in macrophages, including Arg1 and cluster of differentiation 206 (CD206) (304, 305). In addition, enzymes highly expressed by MSCs, such as indoleamine 2,3-dioxygenase (IDO), can deplete tryptophan in the local microenvironment and generate metabolites (e.g., kynurenine), thereby establishing an immunosuppressive milieu that shifts macrophage function toward a tolerant and reparative state (306). A study involving IDO gene-modified MSCs in a model of ischemic acute kidney injury demonstrated that, following renal accumulation of MSCs, macrophages transitioned from the M1 phenotype to the cluster of differentiation 163-positive (CD163^+^) M2 phenotype. This effect was accompanied by a significant decline in pro-inflammatory factor activity and marked reductions in tubular necrosis and fibrosis (307).

MSC-derived exosomes (MSC-Exo) are enriched with informational molecules such as microRNA, proteins, and lipids, which can be internalized by macrophages, thereby altering the functional phenotype of recipient cells. Numerous miRNAs carried by MSC-Exo (e.g., miR-21-5p and miR-146a) downregulate the TLR4/NF-κB pathway or interferon regulatory factor 5 (IRF5) expression in macrophages, thus inhibiting M1 polarization and promoting M2-associated gene expression through genetic regulation (303, 308). In myocardial IRI, administration of MSC-Exo reduces the production of TNF-α and IL-1β by macrophages in the infarct area, upregulates the expression of anti-inflammatory products such as IL-10 and chitinase 3-like protein 3 (Ym1), and facilitates the recovery of cardiac function (302, 309). In renal IRI, treatment with MSC-Exo significantly elevates the expression levels of Arg1 and CD206 in macrophages, while markedly decreasing the degree of inflammatory infiltration and apoptosis rates in renal tissue (303). More importantly, as a cell-free derivative, MSC-Exo offers advantages over MSCs themselves, including easier cryopreservation, scalable preparation, and standardized production. Furthermore, it can be modified through genetic engineering, such as overexpressing specific miRNAs or cytokines, to enhance targeting and strengthen the promotion of macrophage M2 polarization (310).

In addition to MSCs and their exosomes, other cell therapies (e.g., adoptive transfer of regulatory T cells or pre-polarized M2 macrophages) have also shown potential in mitigating IRI damage in certain studies. The core mechanisms involve the paracrine release of anti-inflammatory mediators (e.g., IL-10) and the reprogramming of endogenous macrophage polarization phenotypes (311, 312). However, MSC-related therapies are more suitable for regulating the complex pathological microenvironment of IRI due to their multi-target paracrine effects and favorable safety profile. Therefore, MSC transplantation and exosome-based therapy are regarded as highly promising translational strategies for intervention in IRI. These approaches exerted obvious tissue-protective effects in animal models of multi-organ IRI involving the heart, liver, and kidneys. In addition, the usefulness of these approaches in scenarios such as post-organ transplantation protection and ischemic cardiovascular and cerebrovascular diseases is currently being investigated in exploratory clinical studies.

#### 5.3.2 Nanocarrier delivery systems

The development of nanomedicine has provided novel approaches for the targeted delivery of macrophage polarization modulators. By employing size-controllable and surface-modifiable nanocarriers, active payloads such as drugs and gene fragments can be precisely delivered to the tissue microenvironment where macrophages reside, or even directed into the interior of macrophages. These approaches enhance the efficiency of interventions targeting macrophage polarization, while reducing systemic side effects. Among numerous nanodelivery systems, liposomes represent one of the earliest and most mature carriers. Liposomes exhibit excellent biocompatibility and structural plasticity, enabling specific targeting of macrophages through modulation of surface charge or decoration with targeting ligands (313). In a study of hepatic IRI, the Ras homolog gene (Rho) kinase inhibitor fasudil was encapsulated within modified liposomes. This targeted formulation selectively accumulated in hepatic KCs and monocytes/macrophages, efficiently inhibiting the activity of Rho-associated coiled-coil forming protein kinase 2 (ROCK2) within macrophages, inducing a phenotypic shift from the M1 to M2 phenotype, and reducing the release of pro-inflammatory factors. Ultimately, these effects resulted in significant alleviation of inflammatory damage in hepatic IRI. Moreover, since the drug primarily acted on macrophages, ROCK signaling in systemic vascular smooth muscle remained largely unaffected, avoiding hypotension and other side effects commonly associated with systemic administration (314). In addition to small-molecule drugs, liposomes are also suitable for delivering nucleic acid therapeutics to regulate macrophage gene expression. Studies have demonstrated that neutrophil membrane-camouflaged lipid nanoparticles loaded with TLR4-specific small-interfering RNA (siRNA), when applied in a mouse model of myocardial ischemia–reperfusion, silenced the TLR4 gene in macrophages. The nanoparticles leveraged the inflammation-homing properties of neutrophil membranes to accumulate in damaged myocardium, while efficiently transfecting siRNA into macrophages. These effects successfully downregulated the TLR4/NF-κB signaling pathway, thereby inhibiting M1 macrophage polarization and mitigating myocardial inflammatory necrosis (315, 316).

Polymeric nanoparticles are equally important in delivery systems owing to their material and structural diversity. Carriers exemplified by poly (lactic-co-glycolic acid) nanoparticles can encapsulate various polarization-modulating agents, including M2-promoting factors, antioxidants, and siRNAs or miRNAs that interfere with specific signaling pathways. In studies of autoimmune diseases, poly (lactic-co-glycolic acid) nanoparticles co-delivering RELA-specific siRNA and anti-inflammatory small molecules to macrophages successfully induced a shift from the M1 to M2 phenotype (317). This principle can be extended to IRI scenarios, where co-delivery of inflammation signaling inhibitors and pro-repair factors synergistically regulates macrophage function. Compared with conventional administration methods, polymeric nanoparticles protect encapsulated bioactive substances from rapid degradation and enhance macrophage uptake through targeting modifications, thereby achieving more efficient polarization interventions.

Recently emerged metal-organic framework (MOF) materials have also become a significant component in the nanodelivery field. MOFs, self-assembled from metal ions and organic ligands, form porous crystalline structures with ultrahigh specific surface area and loading capacity, enabling the carriage of gases, small molecules, and biomacromolecules (318). In IRI research, MOFs are frequently employed to deliver gaseous signaling molecules such as carbon monoxide (CO), hydrogen sulfide (H_2_S), or antioxidant enzymes to exert anti-inflammatory and antioxidant effects. In a study, a peroxynitrite-responsive biomimetic nanoplatform was constructed by loading a CO donor into platelet membrane-coated MOFs, achieving precise CO release in ischemic areas. This process led to the protection of cardiomyocytes and inhibition of inflammatory cell activation (319, 320). Another report described neutrophil-like membrane-coated MOFs for the delivery of siRNA targeting NOX4 (a key gene in the macrophage ROS generation pathway) to sites of cerebral IRI. This nanocarrier selectively accumulated in injured macrophages, significantly downregulating NOX4 expression and reducing macrophage-mediated oxidative stress and neuroinflammatory damage (321). These investigations confirmed the unique advantages of MOFs in responsive release within complex microenvironments and macromolecule delivery, making them suitable for the precise modulation of macrophage inflammatory phenotypes. Cell membrane-biomimetic nanoparticles achieve advanced targeting and biological functions by mimicking the surface characteristics of natural cells. For instance, macrophage membrane-coated nanoparticles can disguise themselves as macrophages in circulation to evade immune clearance and competitively neutralize inflammatory mediators such as endotoxins. Consequently, they mitigate the excessive stimulation of KCs by endotoxins in liver transplantation-associated ischemia–reperfusion (322). Platelet membrane coating confers adhesion to damaged endothelium and co-targeting of macrophages, simultaneously promoting reperfusion in ischemic areas and suppressing inflammatory responses (323, 324). By selecting membranes from different cellular sources, such as neutrophil membranes for inflammation homing, erythrocyte membranes for prolonged circulation, or T-cell membranes for tumor infiltration, researchers can tailor the distribution and functionality of nanocarriers in the IRI milieu. In myocardial and cerebral ischemia models, cell membrane-biomimetic nanosystems have demonstrated superior performance over conventional nanoparticles, including higher macrophage targeting, stronger anti-inflammatory effects, and better biocompatibility (323).

In summary, nanodelivery systems provide flexible and diverse platforms for the macroscopic regulation of macrophage polarization. Rational design of liposomes, polymers, MOFs, and other carriers can be combined with biomembrane camouflage techniques. Through this approach, M2-promoting factors, antioxidants, or gene-regulatory molecules can be precisely delivered to macrophages and their microenvironments. These strategies have been linked to reductions in inflammation, promotion of angiogenesis, and improvements in functional prognosis in animal models, holding promise for future development into precise nanotherapies targeting the inflammatory response in IRI.

### 5.4 Targeting tissue-resident macrophages before reperfusion

Notably, tissue-resident macrophages are already present in ischemic organs prior to reperfusion and are among the earliest immune cells to respond to hypoxic stress (20, 325). This highlights their potential as early therapeutic targets (20). Several strategies may be employed to modulate resident macrophages before reperfusion. Preconditioning approaches, including ischemic preconditioning and pharmacological interventions, can induce a protective phenotype (326). In addition, metabolic reprogramming may enhance their resistance to hypoxic stress (327). Furthermore, targeted modulation of pattern recognition receptor signaling could attenuate excessive inflammatory activation upon reperfusion (328).

However, selectively targeting resident macrophages without affecting subsequently recruited monocyte-derived macrophages remains a major challenge and represents an important direction for future research (20).

## 6 Discussion

Macrophages play a dual role in IRI, with their dynamic polarization directly influencing the extent of inflammatory damage and the outcome of tissue repair. This review systematically examined the mechanisms underlying macrophage polarization in IRI, the consequences of its dysregulation, and intervention strategies involving multiple regulatory pathways, such as transcriptional control, metabolic reprogramming, epigenetic modifications, and mechanotransduction signaling. Nevertheless, several critical challenges persist in current research on macrophage polarization. Firstly, there is insufficient understanding of the spatiotemporal dynamics of macrophage polarization states, with the patterns of phenotypic switching across different injury phases and tissue regions remaining unclear. Secondly, although macrophages exhibit organ-specific heterogeneity, organ-tailored intervention protocols are lacking. “One-size-fits-all” immunosuppressive approaches yield limited efficacy, highlighting the shortcomings of overlooking macrophage diversity. Furthermore, reliable biomarkers for monitoring the macrophage polarization status and enabling patient stratification are currently absent. Without such indicators, it is difficult to identify patients likely to benefit from macrophage-targeted therapies or to monitor treatment responses in real time for strategy optimization.

**Figure 8:**
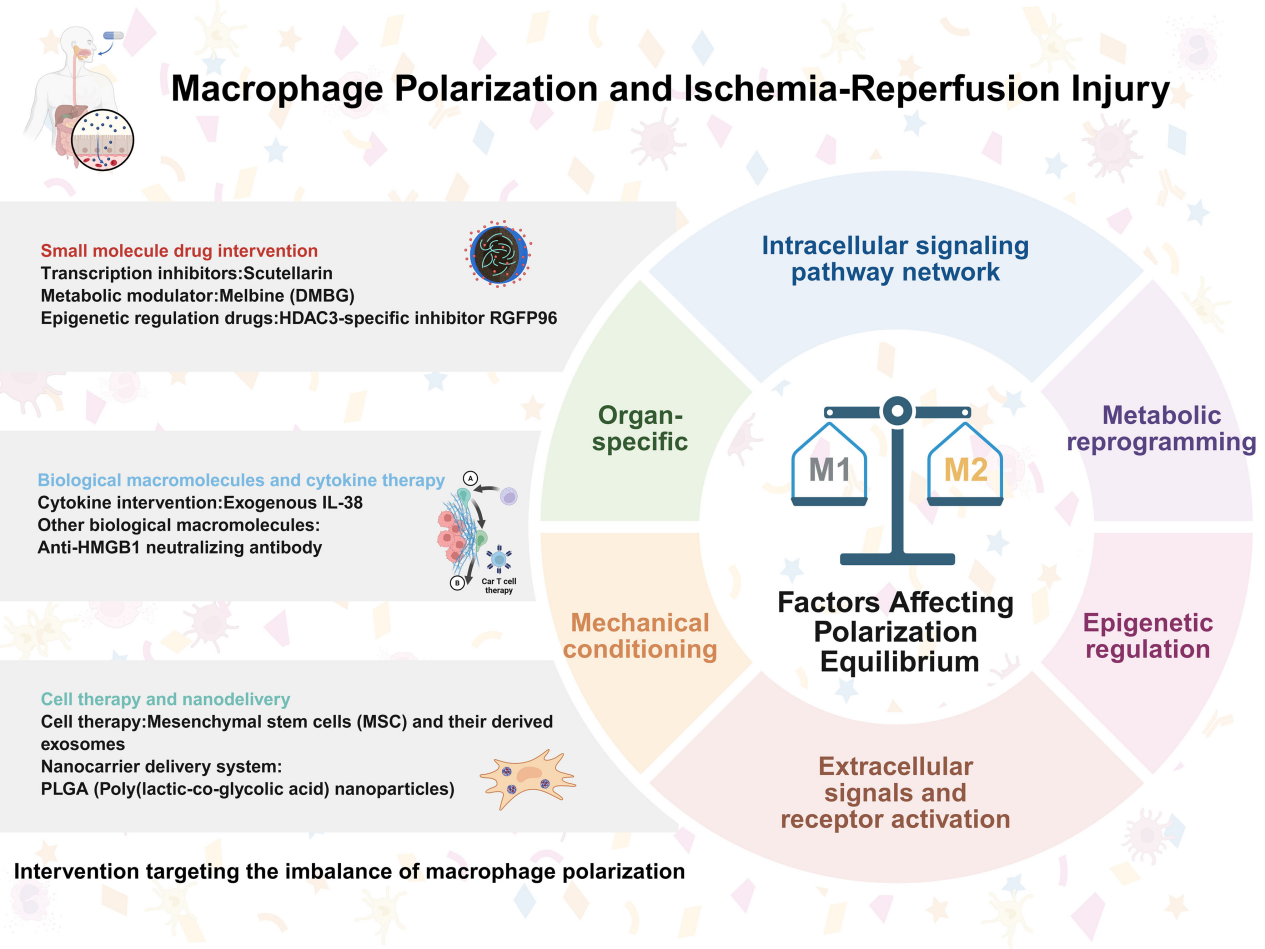
Panoramic view of multifactorial regulation and intervention strategies for macrophage M1/M2 polarization balance in IRI. The balance of macrophage M1/M2 polarization can influence the progression of IRI diseases. Factors such as intracellular signaling pathways, metabolic reprogramming, and epigenetic regulation can regulate the balance of macrophage polarization. The balance of polarization can be restored through intervention measures, such as small-molecule drugs (e.g., baicalin), biological macromolecules/cytokines (e.g., exogenous IL-38), cell therapy and nanodelivery (e.g., mesenchymal stem cell exosomes). IRI, ischemia–reperfusion injury.

To address these challenges, emerging technologies offer opportunities for in-depth dissection of macrophage polarization networks and precise modulation. Techniques such as spatial transcriptomics can resolve the spatial heterogeneity of gene expression *in situ* within tissues, thereby elucidating the specific intercellular interactions and signaling networks in the injured microenvironment. Moreover, the integration of gene editing tools with advanced *in vivo* imaging enables researchers to precisely manipulate macrophage-related genes and track their dynamic changes in living organisms in real time. These advancements are expected to comprehensively elucidate the molecular networks underlying the regulation of macrophage polarization and lay the foundation for personalized precision interventions (**Figure 8**).

In conclusion, the modulation of macrophage polarization balance opens new avenues for the treatment of IRI. There is an urgent need to establish a multi-omics-integrated dynamic intervention framework that precisely regulates macrophage functional states at different stages of injury. This endeavor will accelerate the translation of precision immune modulation from bench to bedside. With deepening insights into polarization imbalance mechanisms and the advent of novel technologies, comprehensive immune management from acute inflammatory damage control to long-term tissue regeneration promotion holds promise for improving outcomes in patients with IRI and offering new hope for affected individuals.

## Glossary

Arg1: arginase 1
ATP: adenosine triphosphate
AKT: Protein Kinase B
ACLY: ATP-citrate lyase
acetyl-CoA: acetyl-coenzyme A
ACOD1: aconitate decarboxylase 1
AP-1: activator protein-1
BBB: blood–brain barrier
CXCL8: C-X-C motif chemokine ligand 8
CCL2: C-C motif chemokine ligand 2
CCR2^+^: C-C chemokine receptor type 2 (CCR2)-positive
CD11b: integrin alpha M
CBP: CREB-binding protein
CTLA-4-Ig: Cytotoxic T-lymphocyte-associated protein 4-immunoglobulin
CD206: cluster of differentiation 206
CO: carbon monoxide
DAMPs: Damage-associated molecular patterns
ERK: extracellular regulated kinase
FAO: fatty acid oxidation
FAK: focal adhesion kinase
HMGB1: high mobility group box 1 protein
HIF-1α: hypoxia-inducible factor-1α
HSPs: heat shock proteins
sirtuins: histone deacetylases
H3K27me3: histone H3 lysine 27 trimethylation
H3K4me3: histone H3 lysine 4 trimethylation
HATs: histone acetyltransferases
H2S: hydrogen sulfide
IRI: ischemia–reperfusion injury
IL-1β: interleukin-1β
IL-1R: interleukin-1 receptor
IKK: inhibitor of κB kinase
IFN-γ: Interferon-γ
iNOS: inducible nitric oxide synthase
IRAK4: interleukin-1 receptor-associated kinase 4
IDH: isocitrate dehydrogenase
IRG1: immune responsive gene 1
IL-1RAPL1: IL-1 receptor accessory protein-like 1
IDO: indoleamine 2,3-dioxygenase
CD163^+^: cluster of differentiation 163-positive
IRF5: interferon regulatory factor 5
JAK: Janus kinase
JMJD3: Jumonji domain-containing protein 3
Keap1: Kelch-like ECH-associated protein 1
KCs: Kupffer cells
LPS: lipopolysaccharide
MAPK: mitogen-activated protein kinase
mTORC1: mammalian target of rapamycin complex 1
MMP-2: matrix metalloproteinase-2
MyD88: myeloid differentiation primary response protein 88
mTOR: mammalian target of rapamycin
Mincle: macrophage-inducible C-type lectin receptor
miRNAs: microRNAs
Metrnl: Meteorin-like
MSCs: mesenchymal stem cells
MSC-Exo: MSC-derived exosomes
MOF: metal-organic framework
NO: nitric oxide
NF-κB: nuclear factor-κB
NLRP3: NOD-like receptor family pyrin domain-containing 3
Nrf2: nuclear factor erythroid 2-related factor 2
NADPH: nicotinamide adenine dinucleotide phosphate
NOX: NADPH oxidase
NADH: nicotinamide adenine dinucleotide
NFAT: nuclear factor of activated T cells
NAMPT: nicotinamide phosphoribosyltransferase
OXPHOS: oxidative phosphorylation
P2X7: P2X purinergic receptor 7
PDGF: platelet-derived growth factor
PPAR-γ: peroxisome proliferator-activated receptor γ
PI3K: Phosphatidylinositol 3-Kinase
Prdx1: peroxiredoxin-1
PHD: Prolyl hydroxylase
PFKFB3: 6-phosphofructo-2-kinase/fructose-2,6-bisphosphatase 3
PGE2: prostaglandin E2
ROS: reactive oxygen species
Rh-GFFYE: Rhein-GFFYE
RBP-Jκ: recombination signal binding protein for immunoglobulin kappa J region
RAGE: receptor for advanced glycation end products
Rho: Ras homolog gene
ROCK2: Rho-associated coiled-coil forming protein kinase 2
STAT1: signal transducer and activator of transcription 1
SOCS1/3: suppressors of cytokine signaling 1/3
Syk: spleen-associated tyrosine kinase
SDH: succinate dehydrogenase
SIRT1: silent information regulator 1
siRNA: small-interfering RNA
TNF-α: tumor necrosis factor-α
TGF-β: transforming growth factor-β
TLR2/4: toll-like receptors 2/4
TNFR1: tumor necrosis factor receptor 1
TREM2: triggering receptor expressed on myeloid cells 2
TRAF6-dependent: tumor necrosis factor receptor-associated factor 6-dependent
TRPV4: transient receptor potential vanilloid subtype 4
TAZ: transcriptional co-activator with PDZ-binding motif
TEAD: TEA domain
TSG-6: tumor necrosis factor-stimulated gene 6
USP14: ubiquitin-specific protease 14
VEGF: vascular endothelial growth factor
YAP: Yes-associated protein
Ym1: chitinase 3-like protein 3
4-OI: 4-octyl itaconate
α-KG: α-ketoglutarate.
